# Tolerant mechanisms to O_2_ deficiency under submergence conditions in plants

**DOI:** 10.1007/s10265-020-01176-1

**Published:** 2020-03-18

**Authors:** Motoka Nakamura, Ko Noguchi

**Affiliations:** 1 Department of Bio-Production, Faculty of Bio-Industry, Tokyo University of Agriculture, 196 Yasaka, Abashiri, Hokkaido 099-2493 Japan; 2 School of Life Sciences, Tokyo University of Pharmacy and Life Sciences, 1432-1 Horinouchi, Hachioji, Tokyo 192-0392 Japan

**Keywords:** Anoxia, Hypoxia, Low O_2_ escape and low O_2_ quiescence strategies (LOES and LOQS), Nitrogen acquisition strategy, Re-oxidative stress, Respiration, Wetland plants

## Abstract

Wetland plants can tolerate long-term strict hypoxia and anoxic conditions and the subsequent re-oxidative stress compared to terrestrial plants. During O_2_ deficiency, both wetland and terrestrial plants use NAD(P)^+^ and ATP that are produced during ethanol fermentation, sucrose degradation, and major amino acid metabolisms. The oxidation of NADH by non-phosphorylating pathways in the mitochondrial respiratory chain is common in both terrestrial and wetland plants. As the wetland plants enhance and combine these traits especially in their roots, they can survive under long-term hypoxic and anoxic stresses. Wetland plants show two contrasting strategies, low O_2_ escape and low O_2_ quiescence strategies (LOES and LOQS, respectively). Differences between two strategies are ascribed to the different signaling networks related to phytohormones. During O_2_ deficiency, LOES-type plants show several unique traits such as shoot elongation, aerenchyma formation and leaf acclimation, whereas the LOQS-type plants cease their growth and save carbohydrate reserves. Many wetland plants utilize NH_4_^+^ as the nitrogen (N) source without NH_4_^+^-dependent respiratory increase, leading to efficient respiratory O_2_ consumption in roots. In contrast, some wetland plants with high O_2_ supply system efficiently use NO_3_^−^ from the soil where nitrification occurs. The differences in the N utilization strategies relate to the different systems of anaerobic ATP production, the NO_2_^−^-driven ATP production and fermentation. The different N utilization strategies are functionally related to the hypoxia or anoxia tolerance in the wetland plants.

## Introduction

O_2_ deficiency in roots is often caused by frequent flooding during rains, submergence by excess rainfall, soil compaction, and increased microorganism activity caused by the rise in temperature. Prolonged submergence of the roots in water can even lead to O_2_ deficiency in shoots. These factors negatively affect the growth and survival of the whole plant, both in natural and agricultural ecosystems. The degree of O_2_ deficiency in plant cells is classified as either hypoxia or anoxia. Hypoxia is characterized by restriction of aerobic metabolism, in which ATP production via the mitochondrial oxidative phosphorylation and NAD^+^ regeneration via the mitochondrial electron transport chain (mETC) are partially restricted. Anoxia is characterized by anaerobic metabolism, in which ATP is supplied solely via glycolysis, mitochondrial oxidative phosphorylation is completely inhibited, and the cellular ATP content is extremely low (Bailey-Serres and Voesenek 2008). Under these stress conditions, plants suffer from impairments that are caused by cell acidification and accumulation of reducing equivalents that lead to the production of reactive oxygen species (ROS) and reactive nitrogen species (RNS) (Hebelstrup and Møller 2015; Turkan 2018). Large amounts of ROS and RNS are produced under re-oxygenated conditions following the post-hypoxic and anoxic stresses, and they can further potentially damage the organelles. Thus, aerobic metabolism is suppressed during the recovery phase from O_2_ deficiency owing to an inhibition of the metabolic functions (Bailey-Serres and Chang 2005; Fukao et al. 2011; Santosa et al. 2007).

Most terrestrial plants such as Arabidopsis, barley and maize, cannot survive long-term O_2_ deficiency and severe anaerobic conditions even though they can survive under short-term stress with their hypoxia and anoxia tolerant responses. Arabidopsis under hypoxic conditions can regenerate NAD^+^ via mETC and fermentation (Bucher et al. 1994; Dolferus et al. 2008; Ismond et al. 2003; Lasanthi-Kudahettige et al. 2007; Narsai and Whelan 2013), but glycolysis cannot continuously function under anoxic conditions (Lasanthi-Kudahettige et al. 2007; Loreti et al. 2005). The responses to O_2_ deficiency in barley and maize have been examined to maintain their high yields at O_2_ deficiency (Tollenaar and Lee 2002). They can oxidize NAD(P)H to NAD(P)^+^ via glycolysis and fermentation to avoid accumulation of reducing equivalents when they are exposed to hypoxia by flooding (Guglielminetti et al 1995, 1999). They can metabolize RNS such as NO to maintain redox states and energy levels in the cytosol and mitochondria under hypoxia conditions (Igamberdiev and Hill 2004; Igamberdiev et al. 2006; Sowa et al. 1998). Maize can also form aerenchyma to aerate O_2_-deficient cells under hypoxic conditions similar to wetland plants (Armstrong and Armstrong 1994; Drew et al. 2000; Evans 2004; Hu et al. 2013). However, these plants cannot tolerate severe anoxia conditions that are caused by prolonged flooding and the following re-oxygenated condition after O_2_ deficiency.

In contrast, wetland plants such as rice can tolerate severe anoxia and the following re-oxygenation conditions due to repeated flooding. This is because they possess high tolerant mechanisms such as advanced regulation of glycolysis in the cytosol, detoxification of ROS and RNS in the mitochondria, and maintenance of ATP production linked to NO detoxification in the mitochondria (Fukao et al. 2011; Huang et al. 2008; Voesenek and Bailey-Serres 2015). They can also acclimate to severely flooded soils through the O_2_ supply from the aerial organ to O_2_-deficient organ. Their strategy is called low O_2_ escape strategies (LOES). Plants with the LOES phenotypes can change their shoots and roots in response to O_2_ deficiency such as shoot elongation, formation of aerenchyma, barriers to radial O_2_ loss (ROL) from the roots, formation of adventitious roots (ARs), and maintenance of gas films on leaf surface (Colmer 2003; Eysholdt-Derzsó and Sauter 2019; Sorrell and Hawes 2009; Winkel et al. 2013; Winkel et al. 2014). Some wetland plants show another strategy, low O_2_ quiescence strategy (LOQS). Plants with the LOQS phenotypes can survive under sever O_2_-deficient conditions where their aerial parts are completely submerged by flooding. They can cease growth and save their carbohydrate reserves until normal growth condition is recovered (Fukao and Bailey-Serres 2008; Fukao et al. 2006). These cellular and tissue level responses to O_2_ deficiency in wetland plants permit their growth and survival under severe anoxic conditions.

Plant roots that are the sites of active nutrient absorption are often exposed to frequent and large fluctuations of O_2_ concentration for short and long periods compared to the other organs. Wetland plants maintain their root activity by the aeration from the aerial organs to roots through the aerenchyma, and the available O_2_ in their roots depends on their abilities of LOES which are enhanced under O_2_-deficient conditions. Many wetland species specialize in NH_4_^+^ utilization in habitats with a predominance of NH_4_^+^. However, the wetland plants with the ability to supply O_2_ from shoots to roots can utilize NO_3_^−^ in addition to NH_4_^+^, because of nitrification in their rhizosphere by active ROL from their root tips (Brix et al. 2002; Kirk and Kronzucker 2005). Moreover, it was recently reported that the root O_2_ consumption strategies related to nitrogen (N) acquisition differ among species with differences in their ability to O_2_ supply to the roots. The differences in strategies are associated with the differences in the O_2_ demand by the aerobic respiration for root growth and N acquisition (Nakamura and Nakamura 2016; Nakamura et al. 2013). The N acquisition traits linked to the O_2_ supply ability could relate to the hypoxia and anoxia tolerance in wild wetland species.

In this review, we summarize the previous findings on regulatory mechanisms of glycolysis, mitochondrial respiratory systems, primary metabolism in maintaining the energy production and homeostasis under O_2_-deficient and re-oxidative stress conditions, and developmental plasticity underlying acclimation to hypoxic and anoxic conditions in terrestrial, wild wetland, and cultivated species. Particularly, we aim to show different molecular level responses and different cellular and whole-plant level strategies between wetland species with long-term tolerance to O_2_-deficient conditions and terrestrial species with only short-term tolerance to the same conditions. Moreover, we summarize the effects of N sources (NH_4_^+^ and NO_3_^−^) on the root respiratory systems of wetland species, and thereafter, we discuss the characteristics of aerobic and anaerobic root respiration in wetland species associated with the utilization strategies of available inorganic N in their rhizosphere for maintaining energy production.

### Regulatory mechanisms of fermentation and glycolysis under oxygen-deficient conditions

Under O_2_-deficient conditions, ATP production in most plants rapidly declines because the electron transfer in the mETC and flux in the tricarboxylic acid (TCA) cycle slow down and the transcripts encoding many of their enzymes are down-regulated (Narsai et al. 2011). Even under low O_2_ conditions, large amounts of energy are required for maintaining the various cellular components, including proteins, for survival (Mustroph and Albrecht 2003). Plants that are tolerate to O_2_ deficiency have a high ATP production ability which is achieved by the enhancement of fermentation and glycolysis. They can regenerate NAD(P)^+^ from NAD(P)H accumulated by slow electron transport to maintain normal redox level under O_2_-deficient conditions. In the first part of this section, we show mechanisms of ATP production and NAD(P)^+^ regeneration through the fermentation and glycolysis. In the latter part of this section, we show the regulations of glycolysis and cytosolic pH by the utilization of pyrophosphate (PP_i_) which can act as a donor of phosphate for various metabolisms similarly to ATP (Shingaki-Wells et al. 2011).

#### Management of energy crisis through fermentation

**Fig. 1:**
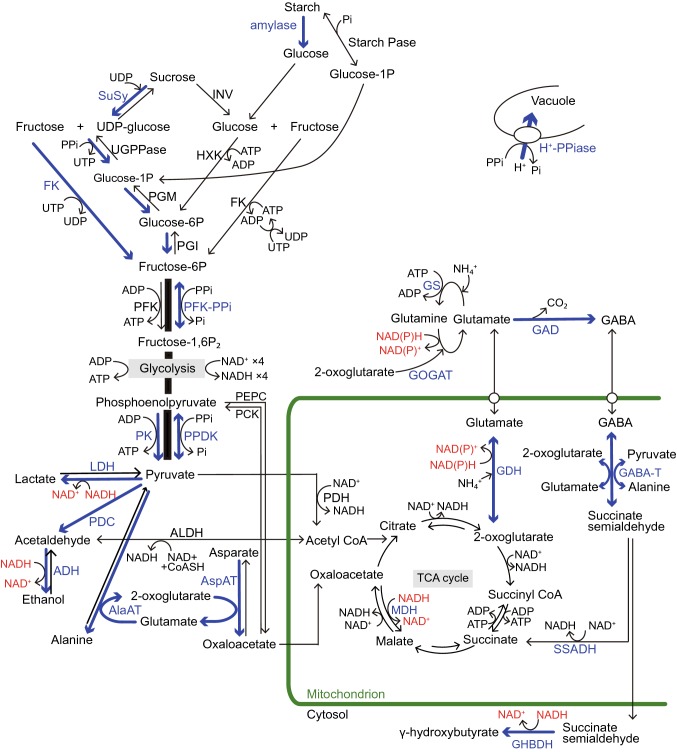
Regulations of sugar catabolism, fermentation, glycolysis, and major amino acid metabolism associated with NAD(P)^+^ regeneration and ATP production in terrestrial and wetland plants under O_2_-deficient conditions. Blue arrows and letters indicate the reactions and enzymes in the up-regulated pathways when the mitochondrial electron transport and the TCA-cycle flux decrease under O_2_-deficient conditions. Red letters indicate the regeneration of NAD(P)^+^ from NAD(P)H. In rice plants, the blue pathways contribute to their tolerance to long-term O_2_ deficiency compared with the terrestrial plants. Some wetland plants such as rice also have a high ability to optimally regulate the pyruvate level by activation of pyrophosphate (PP_i_)-dependent phosphofructokinase (PFK-PP_i_) and pyruvate phosphate dikinase (PPDK) that consume PP_i_ instead of ATP for energy conservation. Besides glycolysis, PP_i_ is consumed to regulate the cytosolic pH by the tonoplast H^+^-pumping pyrophosphatase (H^+^-PP_i_ase) instead of H^+^-ATPase in wetland plants. Although two independent pathways for sucrose degradation contribute to the regulation of glycolytic flux in both terrestrial and wetland plants, the UDP-dependent sucrose synthase (SuSy) pathway is regarded as energetically more advantageous for survival under O_2_-deficient conditions than the invertase (INV) pathway because here, PP_i_ is utilized instead of ATP. Sugar supply to glycolysis through starch mobilization is observed in species with developed storage organs such as tuber, rhizome, and endosperm. In NAD(P)H regeneration during the metabolisms of 2-oxoglutarate and glutamate associated with γ-aminobutyric acid (GABA) production, the glutamate dehydrogenase (GDH) pathway without ATP consumption is more efficient in energy consumption than the NAD(P)H-dependent glutamine: 2-oxoglutarate aminotransferase (GOGAT) pathway with ATP consumption. The accumulation of some amino acids such as GABA, alanine, and glutamate play an important role in avoiding carbohydrate loss not only during O_2_-deficient conditions but also during the recovery phase of re-oxygenation after hypoxia/anoxia. Alanine accumulation by alanine aminotransferase (AlaAT) can operate non-circular TCA-cycle and gluconeogenesis under O_2_ deficiency and re-oxygenation. Abbreviations are as follows: ADH, alcohol dehydrogenase; AlaAT, alanine aminotransferase; ALDH, acetaldehyde dehydrogenase; AspAT, aspartate aminotransferase; CoASH, coenzyme A; FK, fructokinase; GABA-T, GABA transaminase; GAD, glutamate decarboxylase; GHBDH, γ-aminobutyrate dehydrogenase; Glucose-1-P, glucose-1-phosphate; GS, glutamine synthetase; HXK, hexokinase; LDH lactate dehydrogenase; MDH, malate dehydrogenase; PCK, phosphoenolpyruvate carboxykinase; PDC, pyruvate decarboxylase; PDH; pyruvate dehydrogenase; PEPC, phosphoenolpyruvate carboxylase; PFK, ATP-dependent phosphofructokinase; PFK-PPi, PPi-dependent phosphofructokinase; PGI, phosphoglucoisomerase; PGM, phosphoglucomutase; Pi, phosphate; PK, pyruvate kinase; PPDK, pyruvate Pi dikinase; SSADH, succinate semialdehyde dehydrogenase; Starch Pase, starch phosphorylase; TCA, tricarboxylic acid; UDP, uridine diphosphate; UGPPase, UDP-glucose pyrophosphorylase; UTP, uridine triphosphate

Ethanol and lactate fermentation are two major metabolic pathways that produce energy under O_2_-deficient conditions. Pyruvate derived from glycolysis is converted to ethanol through the coupled reactions catalyzed by pyruvate decarboxylase (PDC) and alcohol dehydrogenase (ADH) in the ethanol fermentation pathway, and to lactate by lactate dehydrogenase (LDH) in the lactate fermentation pathway with the concomitant oxidation of NADH to NAD^+^ (Fig. 1). As the glycolytic flux to the ethanol fermentation pathway is higher than that to the lactate fermentation pathway in several plants, ethanol fermentation is considered to strongly contribute to low O_2_ tolerance compared to lactate fermentation (Licausi and Perata 2009). A shift from pyruvate metabolism via the TCA cycle to the ethanol fermentation pathway is attributed to the K_m_ of PDC, which is similar to that of the accumulated pyruvate level under O_2_-deficient conditions (Pronk et al. 1996). In rice plants, this shift is also associated with the inactivation of pyruvate dehydrogenase (PDH) by an up-regulation of PDH kinase (Marillia et al. 2003) and decreased translation of PDH mRNA (Branco-Price et al. 2008).

The ethanol fermentation pathway is controlled by the activity level and gene expression of PDC because the maximum catalytic activity of PDC is low (Drew 1997; Ismond et al. 2003; Mithran et al. 2014; Morrell et al. 1990). PDC-overexpressed terrestrial plants such as Arabidopsis and tobacco were reported to exhibit much higher ethanol concentration in their leaves compared with the wild types, and their survival rates under low O_2_ conditions were enhanced (Bucher et al. 1994; Ismond et al. 2003).

In Arabidopsis plants, during O_2_ deficiency, *PDC1* and *PDC2* are up-regulated in roots and leaves, respectively (Mithran et al. 2014). In rice plants, PDC is induced at both the transcript and protein levels during O_2_ deficiency (Narsai and Whelan 2013). Experiments examining the effects of PDC level on the submergence tolerance of rice had revealed that the correlation between PDC activity or expression and submerge tolerance was stronger in shoots that show high growth than those in roots and endosperms that show low growth at O_2_ deficiency (Rahman et al. 2001). Moreover, rice varieties with high shoot elongation under anaerobic conditions showed active ethanol fermentation due to the high activity and gene expression of PDC in shoots, where the ethanol production was more active than that in roots under dark anaerobic conditions (Mustroph et al. 2006a, b). In contrast, there were no differences in root PDC activities between the varieties, and only a slight increase in activity was observed in submerged tolerant species under severe O_2_ deficiency at night (Mohanty and Ong 2003; Rahman et al. 2001). These results suggest that the distribution range of wild wetland species is characterized by the fermentation abilities of the species due to the PDC activities in their shoots rather than those in their roots, and that the fermentation abilities in roots to survive in hypoxic soils are similar among species.

The activation of ADH does not lead to the acceleration of ethanol production in maize (Roberts et al. 1989). As with terrestrial species, in some rice cultivars, although the expressions of ADH genes (*ADH1* and *ADH2*) are not considered to be major determinants of the seedling vigor during hypoxic stress caused by submergence, these gene expressions respond to low O_2_ stress (Vu et al. 2009). These reports suggest that ADH activity is not crucial in ethanol fermentation for energy compensation for anoxia tolerance. However, increased activity of ADH may be crucial in utilizing ethanol as a carbon (C) source under conditions where the plant experiences different stress at the same time or during re-oxygenation after flooding (Gibbs and Greenway 2003; Tsuji et al. 2003). Moreover, ADH has an essential role in germination and subsequent survival of the seedling at O_2_ deficiency (Rahman et al. 2001).

Lactate fermentation also plays a crucial role in plant survival under anoxic conditions through the activity of LDH that reversibly catalyzes pyruvate and lactate (Fig. 1). Under O_2_-deficient conditions, pyruvate is converted to lactate via LDH, and under re-oxygenated conditions after flooding, the accumulated lactate quickly disappears and the glycolytic flux is regulated by the regeneration of pyruvate from lactate (Germain et al. 1997a). Additionally, as lactate can induce cytosolic acidification and the cytosolic pH is adjusted to an optimal value for the PDC activity, lactate accumulation induces metabolic change from lactate fermentation to ethanol fermentation (Davies 1980). Dolferus et al. (2008) have also reported that increased LDH activity induces ethanol fermentation in Arabidopsis. Excessive accumulation of lactate causes cell death by a sharp decline of the cell pH. Thus, many plants possess lactate efflux mechanism from their cytoplasm. In Arabidopsis, a high cytosolic accumulation of lactate is prevented by lactate excretion via a hypoxia-induced nodulin intrinsic protein NIP2;1 (Choi and Roberts 2007). A similar function was reported in some pleiotropic drug resistance (PDR) type ATP-binding cassette (ABC) transporters, whose expression in rice is regulated by lactate and other weak acids (Moons 2008).

#### Regulation of glycolytic flux via carbohydrate mobilization, sucrose catabolism, and amino acid metabolism under anaerobic conditions

Under anaerobic conditions, glycolysis operates for ATP production through the stable supply of carbohydrate by starch mobilization and sucrose degradation. Moreover, metabolisms of some amino acids such as glutamate, alanine and γ-aminobutyric acid (GABA) lead to the maintenance of glycolysis operation through the NAD(P)^+^ regeneration and the stable preservation of carbohydrates under anaerobic conditions. Under post-anoxic conditions, metabolisms of GABA and alanine contribute to the avoidance from ROS accumulation and the recovery of aerobic metabolism during re-oxygenation, respectively. Terrestrial and wetland plants show different responses in starch mobilization, sucrose degradation and amino acid metabolisms to O_2_ deficiency.

##### Starch mobilization in the maintenance of glycolysis

A crucial point in the maintenance of glycolysis when plants are exposed to O_2_-deficient conditions is the efficient mobilization of the reserved carbohydrates (Dixon et al. 2006; Drew 1997; Gibbs and Greenway 2003; Licausi and Perata 2009; Sato et al. 2002; van Dongen et al. 2004) (Fig. 1). α-amylase and starch phosphorylase (Starch Pase) are induced by low O_2_ and they convert starch to glucose and glucose-1 phosphate (P), respectively. These reactions are observed in storage organs of cereal grains, potato tubers and rhizomes for their germination and subsequent growth (Bucher and Kuhlemeier 1993; Das et al. 2000; Geigenberger 2003). Rice can germinate under low O_2_ conditions due to the expression of α-amylase genes in its aleurone layer. Other terrestrial cereals such as wheat and barley cannot germinate under the same low O_2_ conditions (Das et al. 2000; Guglielminetti et al. 1995, 1999; Narsai et al. 2009; Ricard et al. 1991). Among the three subfamilies of α-amylases genes (*AMY1A*-*C, AMY2A, and AMY3A*-*F*) in rice, *AMY3D* is anoxic-specific (Loreti et al. 2003; Park et al. 2010). Its induction under anoxia is repressed by high sugar levels, but is independent of gibberellin (GA) levels. This is because its promoter region lacks the distinctive cis-acting element that confers GA-responsiveness (Loreti et al. 2003; Park et al. 2010). In contrast, isoforms encoded by *AMY1A* are found in both aerobic and anaerobic seedlings, and *AMY1A* is induced by GA in seedlings under aerobic but not under anoxic conditions (Loreti et al. 2003; Morita et al. 1998). Thus, under aerobic conditions, GA-dependent activation of *AMY1A* maintains high sugar levels in the aleurone layer, and thereby *AMY3D* induction is prevented. The *AMY3D* expression depends on the cis-acting elements in its promoter region, named sugar repression core (*SRC*, Chen et al. 2006), and trans-acting transcription factor (TF), *MYBS1* (Lu et al. 2002). Moreover, *AMY3D* and *MYBS1* have been reported to be activated by the general regulator, sucrose non-fermenting receptor kinase 1A (OsSnRK1), which is promoted by calcineurin B-like interacting protein kinase 15 (CIPK15). They play a role in sugar and energy depletion signaling (Lee et al. 2014; Lu et al. 2002; Lu et al. 2007).

The mobilization of starch for constant carbohydrate supplementation under O_2_-deficient conditions is not common to all plants. Among terrestrial plants, this ability is limited to species with storage organs such as cereal grains and potato tubers (Arpagaus and Brändle 2000; Dixon et al. 2006; Guglielminetti et al. 1995). These plants can prevent starch depletion from their storage organs because they can sense the sugar level in their cells and subsequently down-regulate their metabolic rates (Arpagaus and Brändle 2000; Dixon et al. 2006). The mobilization of starch and regulation of sugar level at the germination and seedling growth stages have been poorly understood in wetland species other than rice plants. This metabolism may act as a crucial tolerance mechanism in the germination and early development of wetland species because many wetland species with relatively large endosperms (members from the Gramineae and Cyperaceae families) (Kettenring and Galatowitsch 2007; Leck and Brock 2000; Wijte and Gallagher 1996) and developed rhizomes (members from the Nymphaeaceae and Menyanthaceae families) can grow in stagnant soil with low O_2_ condition.

##### Two independent pathways for the sucrose degradation

The bidirectional UDP-dependent sucrose synthase (SuSy) and the unidirectional invertase (INV) are two distinct pathways for degradation of sucrose in plant cells (Fig. 1). SuSy consumes a net of one mol PP_i_ per one mol sucrose when UDP glucose and fructose are substrates for glycolysis. This is because the by-product of UDP glucose pyrophosphorylase, UTP, is used for the formation of phosphorylated fructose by fructokinase (FK), and simultaneously ATP is regenerated from ADP via NDP kinase (Bailey-Serres and Voesenek 2008; Guglielminetti et al. 1999). In contrast to the consumption of PP_i_ by SuSy, the INV reaction involves two mols ATP per mol sucrose (Mustroph et al. 2005). Therefore, SuSy is regarded as a more energetically advantageous pathway in various species for survival under O_2_-deficient conditions than INV. Indeed, transgenic potato tubers with elevated INV activity were unable to maintain ATP levels under low O_2_ conditions (8% O_2_) (Bologa et al. 2003).

Responses of activities and transcriptions to low O_2_ conditions differ between SySy and INV. The activity and mRNA transcript level of SuSy are rapidly increased by sugar starvation, in contrast to the constitutive expression of INV in various terrestrial and wetland plants (Branco-Price et al. 2008; Koch 2004; Lasanthi-Kudahettige et al. 2007; Loreti et al. 2005). Comparative analysis of gene inductions and protein expressions with or without sucrose addition revealed that SuSy gene expression is up-regulated by sensing sugar starvation as signals (Contento et al. 2004; Liu et al. 2010; Loreti et al. 2005; Nicolai et al. 2006; Rolland et al. 2006). In rice, six SuSy isoforms localized in roots, mesophylls, and phloem are tissue-specifically expressed at different developmental stages (Hirose et al. 2008; Wang et al. 1999). Particularly, the expression of *SUS2* significantly increases in germinating seeds and growing seedlings under anoxic conditions (Hirose et al. 2008), indicating that *SUS2* can serve not only as a housekeeper but also as the initial reaction of sucrose degradation during stress. In addition to the single hypoxia-inducible SuSy isoform, multi-expressions of the SuSy isoforms with functional redundancy are required to ensure low O_2_ tolerance in several species of both terrestrial and wetland plants (Bieniawska et al. 2007; Hirose et al. 2008; Wang et al. 1999).

INV catalyzes sucrose into fructose and glucose, which are then phosphorylated by hexokinase (HXK) or FK to be channeled into the glycolytic pathway (Licausi and Perata 2009) (Fig. 1). The glycolytic flux is also regulated by the activation of the INV pathway, but the main pathway of sucrose degradation under aerobic conditions may be the SuSy pathway (Fig. 1). This regulation of glycolysis is noted not only in the roots of terrestrial crops such as maize and tomato (Bouny and Saglio 1996; Germain et al. 1997b) but also in the seedlings of rice (Cho et al. 2006; Guglielminetti et al. 2006). In rice plants exposed to anoxia, *OsFK2* and *OsHXK7* are induced by sensing sucrose starvation as signals (Cho et al. 2006; Guglielminetti et al. 2006; Lasanthi-Kudahettige et al. 2007). Other isoforms, *OsHXK5* and *OsHXK6*, dual-targeted to the mitochondria and nucleus, also act as glucose sensors (Cho et al. 2009; Narsai and Whelan 2013). They directly regulate the downstream factors including CIPK15, which in turn regulate the representative *α*-*AMY3* gene (*RAMY3D*) and *ADH2* expression in rice under low O_2_ conditions (Yim et al. 2012).

##### Metabolism of typical amino acids linked to glycolysis regulation under O_2_-deficient conditions

At low O_2_, NAD(P)^+^ regeneration can be achieved by amino acid metabolisms such as the metabolism of 2-oxoglutarate and glutamate associated with the production of GABA (Fig. 1). The synthetic pathway of GABA through the glutamate decarboxylation by glutamate decarboxylase (GDC) with H^+^ consumption can contribute to the counteraction of cytosolic acidification caused by anoxic stress (Aurisano et al. 1995). The metabolism of 2-oxoglutarate and glutamate is promoted through the glutamine synthetase (GS)-glutamine oxoglutarate aminotransferase (GOGAT) pathway or the glutamate dehydrogenase (GDH) pathway. In the former, GS and GOGAT catalyze the conversion of glutamine to glutamate with 2-oxoglutarate incorporation, whereas in the latter GDH reversibly catalyzes the reaction between 2-oxoglutarate and glutamate (Narsai et al. 2009; Rocha et al. 2010; Shingaki-Wells et al. 2011) (Fig. 1). The GDH pathway does not consume ATP in the conversion of 2-oxoglutarate to glutamate, while the GS-GOGAT pathway consumes one ATP mol per glutamate mol for the conversion of glutamine to glutamate (Gibbs and Greenway 2003) (Fig. 1). Therefore, the GDH pathway is more efficient in energy consumption. Moreover, increased glutamate can act as an amino group donner in the aspartate transamination by aspartate aminotransferase (AspAT) for the production of oxaloacetate, an intermediate product in the TCA cycle during anoxia (Fig. 1). Oxaloacetate is then converted to malate by malate dehydrogenase with NAD(P)^+^ regeneration (Bailey-Serres and Voesenek 2008). Glutamate is simultaneously incorporated into the pathway by alanine aminotransferase (AlaAT) (Bailey-Serres and Voesenek 2008; Ricoult et al. 2006) (Fig. 1). Under hypoxia, strong induction of mRNA levels and enzymatic activities of AspAT, AlaAT, and GDH in the cytosol and mitochondria have been reported in Arabidopsis and rice plants (Klok et al. 2002; Lasanthi-Kudahettige et al. 2007; Liu et al. 2005; Loreti et al. 2005; Mustroph et al. 2010; Narsai and Whelan 2013; Narsai et al. 2011). Thus, the synthesis of these amino acids may contribute to the regulation of glycolysis through NAD(P)^+^ regeneration in both terrestrial and wetland species.

Metabolisms of GABA and alanine seem to play important roles for plants in survival during low O_2_ stress and re-oxygenation. Kreuzwieser and Rennenberg (2014) have reported that the supply of carbohydrates to amino acid metabolism in shoots and roots is a key process that helps plants survive hypoxia. The metabolism of these amino acids does not result in carbohydrate loss compared with fermentation. In Arabidopsis, the importance of organ-specific response in these advantageous amino acid metabolisms has been pointed out. High GABA accumulation in roots was observed under hypoxia compared with that in shoots, while alanine accumulation was observed in both organs (Mustroph et al. 2014). Transcriptome and metabolome analyses of flooding-intolerant Arabidopsis and flooding-tolerant rice and poplar exposed to anoxia indicated that the accumulation of alanine and succinate and the increased activities of fermentation enzymes were observed in all the species, but that the transcriptional regulations of amino acid metabolism and anaerobic fermentation were different among species (Narsai and Whelan 2013; Narsai et al. 2011). Plant species may have specific mechanisms for signal transduction and post-transcriptional regulation in the amino acid and carbohydrate metabolisms in roots and shoots.

The increased accumulation of GABA under anoxia is metabolized by GABA transaminase (GABA-T) to succinic semialdehyde coupling with consumption of 2-oxoglutarate and additional conversion of pyruvate to alanine (Fig. 1). In the subsequent reaction, succinic semialdehyde is converted either to γ-hydroxybutyrate by the NAD(P)H-consuming reaction catalyzed by GABA dehydrogenase (GHBDH) or to succinate that can be channeled to the TCA cycle by succinate semialdehyde dehydrogenase (SSADH) by the NAD^+^-consuming reaction (Fig. 1). Although the high activity of SSADH with NAD^+^ consumption is thought to be disadvantageous for glycolytic regulation under hypoxia, this enzyme can function as one component of the bypass of the TCA cycle (GABA-shunt) to decrease ROS accumulation under re-oxygenation conditions (Bouche et al. 2003) (Fig. 1). Indeed, higher accumulation of SSADH protein was observed under re-oxygenation conditions in Arabidopsis (Bouche et al. 2003).

Besides anoxic stress, post-anoxic stress can also severely damage plant growth owing to large amounts of ROS that are produced in the cells. Particularly, wetland plants such as rice plants often suffer from post-anoxic stress after frequent floods; they have been reported to exhibit significant transcript reprogramming, which rapidly increased the expression of genes encoding TCA-cycle enzymes and levels of metabolites including citrate and 2-oxoglutarate to restore aerobic growth under post-anoxic conditions (Narsai et al. 2009). Moreover, large amounts of alanine generated by AlaAT under anaerobic conditions help plants to survive under subsequent re-oxygenation conditions because alanine can be transported through the xylem as a transportable energy source (De Sousa and Sodek 2003). AlaAT can convert the transported alanine into pyruvate, which can be used in gluconeogenesis or metabolized to acetyl-CoA (Fig. 1); both processes are important for aerobic metabolism during re-oxygenation (Rocha et al. 2010; Shingaki-Wells et al. 2014). In contrast, coleoptiles of anoxia-intolerant wheat seedlings cannot accumulate alanine when they are subjected to anoxia (Shingaki-Wells et al. 2011). These findings suggest that alanine accumulation by activated AlaAT in the flood-tolerant species plays an important role in their survival not only during hypoxia but also during the recovery phase of re-oxygenation after hypoxia.

##### Utilization of available PP_i_ as the phosphate donner instead of ATP

It has been assumed that PP_i_ is particularly favored as a phosphoryl donor compared with ATP in anoxic tissues where the cytosol is acidic (Davies et al. 1993; Felle 2005). Thus, PP_i_ can serve an alternative energy source instead of ATP, and is utilized to maintain the glycolysis flux and regulation of cytosolic pH under low O_2_ conditions where ATP levels are low.

In glycolysis, the enzymes with reversible reactions, PP_i_-dependent phosphofructokinase (PFK-PP_i_) and pyruvate phosphate dikinase (PPDK), can function instead of ATP-dependent phosphofructokinase (PFK-ATP) and pyruvate kinase (PK), respectively (Fig. 1). As PFK-PP_i_ can catalyze fructose 6-P without consumption of ATP, the PFK-PP_i_ function can increase the net ATP production in anoxia-tolerant plants (Huang et al. 2008; Plaxton and Podestá 2006) (Fig. 1). In rice seedlings, the enzymatic activity of PFK-PP_i_ is dramatically increased by 15-fold after 24 h in anoxia (Gibbs et al. 2000; Kato-Noguchi 2002; Mertens et al. 1990). The expression of annotated PFK-PP_i_ genes in anoxic rice coleoptiles is complex. Some are down-regulated and others are up-regulated (Lasanthi-Kudahettige et al. 2007). In contrast, gene expression and protein amounts of the cytosol-type and plastid-type PPDKs in rice are up-regulated under anoxia. Especially the expression of cytosolic-type PPDK is up-regulated by 365-fold when the plants are exposed to anoxia (Lasanthi-Kudahettige et al. 2007; Moons et al. 1998; Shingaki-Wells et al. 2011) and is higher in roots than that in shoots (Huang et al. 2008). This suggests that roots have a higher ability to enhance anoxia tolerance than shoots because roots often experience more frequent fluctuations of O_2_ concentration than shoots.

Induction of PFK-PP_i_ and PPDK is controlled by the cytosolic PP_i_ content under short- and long-term anoxia, but not by the exogenous substrates such as starch and sucrose (Huang et al. 2008). While the PFK-ATP activity is rate-limiting for glycolysis in short-term anoxia, PFK-PP_i_ can compensate for the ATP limitation by using PP_i_. In such a situation, PP_i_ can be provided by a reaction cycle catalyzed by both PPDK and PK. This would accelerate the glycolytic flux and supply energy for survival at the early phase of anoxia. In contrast, if the plants are exposed to long-term anoxia, glycolysis may need to be down-regulated to conserve carbohydrates. Thus, PFK-PP_i_ and PPDK may regulate the PP_i_ level to slow down the net glycolytic flux for survival under long-term anoxia, and thereby the direction of glycolysis is changed to gluconeogenesis. Some reports indicate that this functional regulation of PP_i_ level may strongly contribute towards maintaining glycolysis under severe anoxic conditions where the ethanol fermentation is declined (Colmer et al. 2001; Huang et al. 2008; Kato-Noguchi 2002; Loreti et al. 2005). Therefore, enzyme-mediated reactions can contribute to low O_2_ tolerance in either direction, towards glycolysis or gluconeogenesis, although it is difficult to experimentally show the reaction directions by PFK-PP_i_ and PPDK because of their small free energy values (ΔG) (Huang et al. 2008).

Gene expressions of PFK-PP_i_ and PPDK in anoxia-intolerant Arabidopsis did not show significant changes in anoxia (Lasanthi-Kudahettige et al. 2007; Loreti et al. 2005). Under these conditions, the gene encoding PFK-PP_i_ was up-regulated by 1.9-fold, and PPDK by only 1.1-1.7-fold. This change in PPDK in Arabidopsis was much less than that in the cytosolic-type PPDK in rice plants. In rice plants, PFK-PP_i_ and tonoplast H^+^-PP_i_ase were induced during phosphate (P_i_) deficiency, but the change in PPDK during P_i_ deficiency is unclear (Plaxton 2004). Interestingly, even under various stress environments where the levels of the nucleoside triphosphate (NTP) pools including P_i_, ATP, and ADP were significantly decreased, the PP_i_ levels were relatively stable in rice plants (Plaxton 2004). Especially the PP_i_ concentrations in coleoptiles and cultured cells of rice plants were similar between anoxic and aerated conditions (Kato-Noguchi 2002; Mohanty et al. 1993). These results suggest that the stable level of PP_i_ in rice plants supports a stable response to the crisis in energy production by a sudden O_2_ decrease via PP_i_-dependent enzymes. Moreover, the gene expression of inorganic pyrophosphatase (PP_i_ase) in rice coleoptile was significantly down-regulated by 35-fold when they were exposed to anoxic conditions, whereas the gene expression of inorganic PP_i_ases in Arabidopsis was unchanged or slightly up-regulated under anoxic conditions (Lasanthi-Kudahettige et al. 2007). Consequently, in rice plants under anoxic conditions, PP_i_ is not degraded by inorganic PP_i_ase and large amounts of PP_i_ can be used for the other essential processes (Huang et al. 2008). In contrast, in Arabidopsis cells under anoxic conditions, the PP_i_ content decreases and the plants suffer severe energy deficiency because solute transport across the tonoplast decreases and the cytosol pH is acidic, resulting in ultimately cell death (Fukao and Bailey-Serres 2004). The roles of PP_i_-dependent enzymes in wild wetland plants, other than rice plants, are restricted to a few studies in which the gene expression of cytosolic PPDK in *Eleocharis vivipara* and the activity and induction of PFK-PP_i_ and PPDK in *Potamogeton pectinatus* were examined (Dixon et al. 2006; Summers et al. 2000). As these amphibious plants grow under various O_2_ conditions from land to deep-water wetland, it is assumed that many wild wetland plants may commonly utilize PP_i_-dependent enzymes in response to changes in the O_2_ availability.

Besides glycolysis, many plants consume PP_i_ to regulate the cytosolic pH under O_2_-deficient conditions by the tonoplast H^+^-pumping pyrophosphatase (H^+^-PP_i_ase) instead of H^+^-ATPase (Fig. 1). This can support the essential process of pH regulation even under ATP-deficient conditions. Anoxia-tolerant rice can suppress the pH decline in anoxia to half of that in the normal conditions, while anoxia-intolerant wheat and Arabidopsis suffer severe pH decline during anoxia (Lasanthi-Kudahettige et al. 2007; Loreti et al. 2005; Menegus et al. 1991). In rice plants, the activity of the tonoplast H^+^-PP_i_ase was increased by 75-fold after 6 days in anoxia (Carystinos et al. 1995), and the gene (*Os02g55890*) encoding H^+^-PP_i_ase was up-regulated by 35-fold in anoxia (Lasanthi-Kudahettige et al. 2007).

### Mitochondrial metabolic adaptation to O_2_ deficiency

**Fig. 2:**
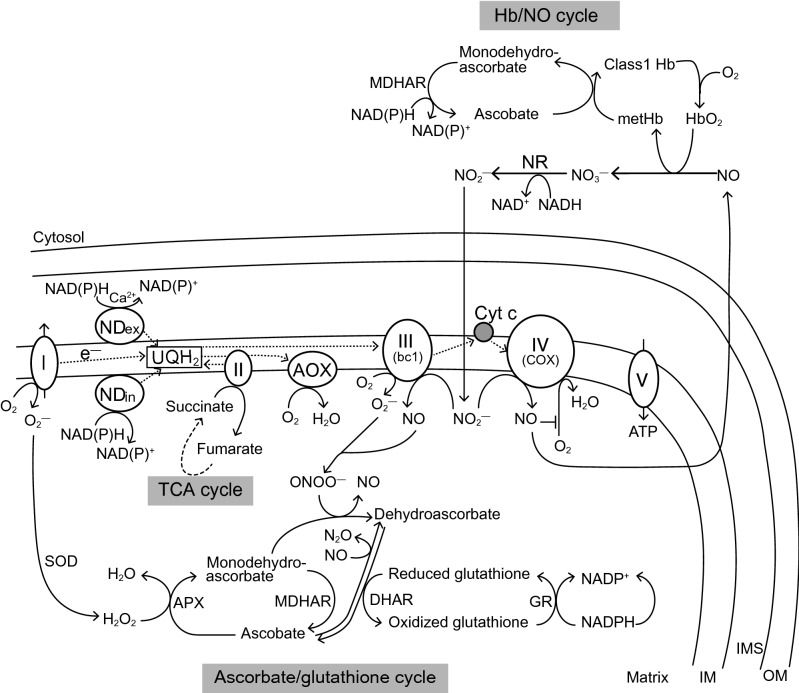
Production and elimination of reactive oxygen species (ROS) and reactive nitrogen species (RNS) in mitochondria and cytosol. H_2_O_2_, NO, O_2_^·−^, and ONOO^−^ produced under hypoxic stress conditions are detoxified by the mitochondrial electron transport chain (mETC) and ascorbate/glutathione cycle in the mitochondrial matrix to maintain a redox balance in the cells. The alternative oxidase (AOX) and type II NAD(P)H dehydrogenases (NDs), ND_ex_, and ND_in_ (NDs located at the outer and inner surfaces of the mitochondrial inner membrane, respectively), can consume the accumulated reducing equivalents for maintaining the mitochondrial homeostasis. AOX has lower affinity to O_2_ than cytochrome *c* oxidase (COX, complex IV); NDs, especially Ca^2+^ dependent ND_in_, have lower affinity to NAD(P)H than complex I and nitrate reductase (NR). In NO scavenging under hypoxic stress condition, ascorbate can contribute to the reduction of NO to N_2_O in the mitochondrial matrix. Ascorbate is converted to monodehydroascorbate by ascorbate peroxidase (APX), which also involves the scavenging of ONOO^−^ and converting it into NO, The NO generated is resupplied to mETC. Ascorbate can also participate in Class 1 hemoglobin (Class 1 Hb) regeneration from methemoglobin (metHb) in the cytosol. Abbreviations are as follows: Cyt c, cytochrome c; DHAR, dehydroascorbate reductase; GR, glutathione reductase; IM, inner membrane; IMS, inter-membrane space; MDHAR, monodehydroascorbate reductase; NO, nitric oxide; NR, nitrate reductase; OM, outer membrane; SOD, superoxide dismutase; TCA, tricarboxylic acid; UQH_2_, ubiquinol

ROS (e.g., H_2_O_2_ and O_2_^·−^) and RNS (e.g., NO and ONOO^−^) can function as important physiological regulators of the intercellular signaling pathway in plant cells (Desikan et al. 2001; LiQiang 2011; Nie et al. 2006). However, they can cause disorders of oxidative phosphorylation due to oxidation and nitration of proteins. Anoxic and post-anoxic stresses by frequent flooding lead to ROS formation due to over-reduction of mETC, and these further lead to decreases in energy production (Blokhina and Fagerstedt 2010; Blokhina et al. 2000; Santosa et al. 2007; Szal et al. 2003). Under such stress conditions, non-phosphorylating components of mETC, the alternative oxidase (AOX) and type II NAD(P)H dehydrogenases (NDs) can consume the accumulated reducing equivalents for maintaining the mitochondrial homeostasis. These components are not coupled with the proton motive force (Blokhina et al. 2014; Maxwell et al. 1999; Millar et al. 2004; Møller 1997; Sweetlove et al. 2006; Szal et al. 2003; Xu et al. 2011) (Fig. 2). The AOX directly transfers an electron from ubiquinol (UQH_2_) to O_2_, and functions in the stress response (Fig. 2). AOX has lower affinity for O_2_ than cytochrome *c* oxidase (COX, complex IV) (Gupta et al. 2009), but O_2_ consumption through AOX does not depend on O_2_ concentration. In contrast, O_2_ consumption through COX decreases depending on the decrease in O_2_ concentration (Zabalza et al. 2009). Thus, AOX can consume reducing equivalents under low O_2_ conditions even when COX activity is inhibited. The AOX activity is controlled by its protein amount, AOX and ubiquinone (UQ) redox states, and pyruvate level (Day and Wiskich 1995; Møller 2001; Simons and Lambers 1999; Vanlerberghe and Mclntosh 1996; Vanlerberghe et al. 2002). A study in which the transcript responses to low O_2_ between flood-tolerant rice and poplar and flood-intolerant Arabidopsis were compared revealed differences in the response of their AOX genes. The induction of AOX in response to low O_2_ was observed in Arabidopsis but not in rice and poplar (Narsai et al. 2011). In the case of Arabidopsis, the expression of AOX gene is induced by citrate accumulation resulting from the inhibition of aconitase activity by NO formed under low O_2_ stress. Consequently, the primary metabolism shifts to amino acid biosynthesis to counteract the energy crisis under low O_2_ stress (Gupta et al. 2012). However, when O_2_ availability is a limiting factor for the O_2_ consumption, this AOX induction would be futile for O_2_ consumption and energetically burdensome. Thus, the inability of Arabidopsis to prevent the induction of AOX genes under low O_2_ conditions could be reasonable for its intolerance to anaerobic conditions (Shingaki-Wells et al. 2014). Interestingly, the *in vivo* AOX activity correlates with the relative growth rate in some wild species (Millenaar et al. 2001). It has been reported that AOX in illuminated leaves can contribute to optimizing the photosynthetic electron transport through the dissipation of excessive reducing equivalents under stress conditions, and the *AOX1* gene expression and AOX capacity are often induced by the presence of exogenous H_2_O_2_ or under stress conditions with high light or high temperatures (Amor et al. 2000; Feng et al. 2010; Murakami and Toriyama 2008; Vanlerberghe and Mclntosh 1996; Vishwakarma et al. 2015; Wagner and Krab 1995; Xu et al. 2011). These results support that AOX is indispensable for the flexible control of ATP synthesis to maintain homeostasis and growth through the interaction between mitochondria and other organelles under various stress conditions (Hansen et al. 2002).

It has been reported that the induction of AOX is associated with the mitochondrial retrograde signaling and AOX can directly influence mitochondrial signaling by decreasing the ROS and uncoupling the electron transport from ATP synthesis (Rhoads and Subbaiah 2007). One of the TFs involving the AOX gene expression is related to abscisic acid (ABA), ABI4; it was reported to be a strong repressor of AOX expression in leaves of Arabidopsis exposed to high light and temperature stress (Giraud et al. 2009; Møller and Sweetlove 2010; Neill et al. 2003; Selinski et al. 2018; Xu et al. 2011). ABI4 is also intimately involved in sugar and plastid retrograde signaling pathways (Woodson and Chory 2008). Therefore, AOX could be controlled through ABI4 to integrate the mitochondrial retrograde signaling and respiratory regulation with other cellular anterograde and retrograde regulatory pathways. Vanlerberghe et al. (2009) indicated that AOX could buffer cellular signaling pathways, including cell death pathways, against adverse conditions. Moreover, AOX can regulate the gene expression of ROS-scavenging enzymes such as glutathione S-transferase, catalase, ascorbate peroxidase (APX), and superoxide dismutase (Giraud et al. 2009; Rhoads and Subbaiah 2007). In many plants under post-anoxic conditions with high ROS production, the AOX induction was found at both the transcript and protein levels to support a rapid response to re-oxygenation shock (Howell et al. 2007; Millar et al. 2004; Narsai et al. 2009).

Ca^2+^-dependent NDs located at the outer (ND_ex_) and inner (ND_in_) surfaces of the mitochondrial inner membrane are not coupled with the generation of the proton motive force, and function as a bypass of complex I (Michalecka et al. 2003; Møller 1997; Rasmusson et al. 2004) (Fig. 2). The ND_ex_ can mainly utilize the cytosolic reducing equivalents (NAD(P)H) and have a higher K_m_ for NADH than those of complex I and nitrate reductase (NR) (Møller et al. 1993). As ND_ex_ can utilize NAD(P)H independently of the other processes of mETC, it can regulate the NAD(P)H levels inside the intermembrane space and cytosol (Igamberdiev and Hill 2004) (Fig. 2). Moreover, it can be regulated by the elevated concentration of cytosolic Ca^2+^, due to Ca^2+^ release from the mitochondria during hypoxia (Fig. 2). An increase in the cytosolic Ca^2+^ concentration is stimulated by NO and H^+^/Ca^2+^ antiport, which is linked to the decrease in the cytosolic pH (Igamberdiev and Kleczkowski 2003; Subbaiah et al. 1998). The increase in Ca^2+^ concentration under anaerobic conditions functions as a signal for the regulation of many enzymes such as GDC and NAD^+^ kinase (Igamberdiev and Hill 2008). Interestingly, *NDB2*, a gene encoding the ND_ex_, is strongly co-expressed with *AOX1a* in Arabidopsis because these genes share many common cis-acting regulatory elements (CAREs) in their promoter regions and are affected in a similar manner (Clifton et al. 2005; Elhafez et al. 2006). Further, some of the NDs (NDC1 and NDA1) are dual-targeted to plastids and peroxisome, therefore, their regulation is affected by the proteins outside the mitochondria (Ho et al. 2008). These indicate that important mitochondrial components involved in stress responses could provide the means for coordinating the activities between the organelles via coregulation and dual localization.

### Effect of inorganic N sources on respiration in plants under the O_2_-deficient conditions

Plant roots play an important role in the absorption and assimilation of N and other essential minerals using respiratory energy. In soil, NO_3_^−^ and NH_4_^+^ are found as inorganic N sources, and the energy cost for NH_4_^+^ assimilation is lower than that for NO_3_^−^ (Bloom et al. 1992). Many terrestrial plants prefer to NO_3_^−^ as inorganic N source, while wetland species specialize in NH_4_^+^ utilization because NH_4_^+^ predominates in flooded soils in their habitats. However, some wetland plants with the ability to supply O_2_ from the shoots to the roots can utilize NO_3_^−^ because active radial O_2_ loss (ROL) from their root tips allow nitrification in their rhizosphere (Brix et al. 2002; Kirk and Kronzucker 2005). The preference of the roots for inorganic N sources affects the ATP production levels and O_2_ concentrations in roots. This is because the respiratory system has different responses to NO_3_^−^ and NH_4_^+^ under anaerobic conditions.

#### Two NO_3_^−^ reduction pathways in plants under O_2_-deficient conditions

**Fig. 3:**
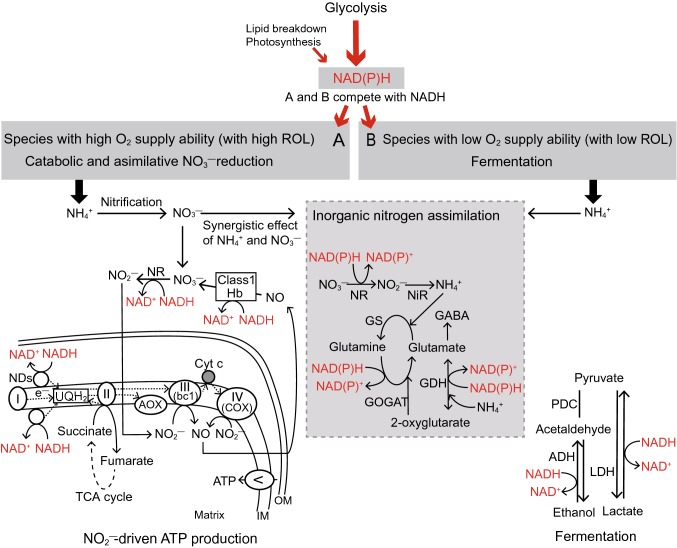
Different utilization strategies of inorganic nitrogen (N) source for the maintenance of ATP production caused by the difference in the O_2_ supply ability in wetland plants under O_2_-deficient condition. NAD(P)H produced mainly during glycolysis, lipid breakdown, and photosynthesis is oxidized to NAD(P)^+^ by the following two pathways competing for the oxidation, assimilation, and catabolic reduction of NO_3_^−^: NO_2_^−^-driven ATP production (A) or fermentation (B). The oxidation of NAD(P)H is shown by red letters and arrows. (A) As the species with high O_2_ supply ability can accelerate nitrification in their rhizosphere by high radial O_2_ loss (ROL) from the roots, they can utilize NO_2_^−^ produced from NO_3_^−^ by hypoxia-induced nitrate reductase (NR) as the electron acceptor in the mitochondrial electron transport chain (mETC) instead of O_2_. NO_2_^−^-driven ATP production enables NAD(P)H oxidation for regulating glycolysis, avoiding cytosolic anoxia, and anaerobic ATP synthesis, which is in the same order as that in the ATP through fermentation during hypoxia. Moreover, species with high potential for NR can oxidize NAD(P)H for N assimilation, and these species can acquire a large amount of N and productivity by the “synergistic effect of NH_4_^+^ and NO_3_^−^”. (B) The species with low O_2_ supply ability specializing in the assimilation of NH_4_^+^ that dominates the anaerobic soil may oxidize NAD(P)H through fermentation. NAD(P)H levels in the A and B pathway are regulated by glycolysis with pyrophosphate (PP_i_) utilization by PP_i_-dependent phosphofructokinase (PFK-PP_i_) and pyruvate phosphate dikinase (PPDK) instead of ATP and metabolisms of major amino acids such as the alanine, glutamate, 2-oxoglutarate, and γ-aminobutyric acid (GABA). Thus, in wetland plants, A and B pathways function as the N utilization strategy in maintaining the ATP production under anaerobic conditions. Abbreviations are as follows: ADH, alcohol dehydrogenase; AOX, alternative oxidase; bc1, cytochrome bc_1_; Class 1 Hb, class 1 hemoglobin; COX, cytochrome *c* oxidase; Cyt c, cytochrome c; GS, glutamine synthetase; GOGAT, glutamine oxoglutarate aminotransferase; GDH, glutamate dehydrogenase; IM, inner membrane; LDH, lactate dehydrogenase; NDs, mitochondrial NAD(P)H dehydrogenases; NiR, nitrite reductase; OM; outer membrane; PDC, pyruvate decarboxylase; TCA, tricarboxylic acid; UQH_2_, ubiquinol, I–V; mitochondrial complexes I–V

Exogenous NO_3_^−^ can act as a terminal acceptor of electrons and protons in the absence of molecular O_2_. NO_3_^−^ can accept reducing equivalents to regenerate NAD(P)^+^ and prevent deteriorative effects of the cytoplasmic acidification through assimilative or catabolic NO_3_^−^-reduction pathways (Fig. 3, Fan et al. 1997; Müller et al. 1994; Vartapetian and Polyakova 1999). NAD(P)H can be oxidized by the assimilative pathway in which NO_3_^−^ is reduced to NO_2_^−^ and NH_4_^+^, and by the catabolic pathway involving the reductive NO_2_^−^-dependent NO production. These two pathways contribute to up-regulation of glycolysis under hypoxic and anoxic conditions due to the facilitation of glycolytic flux (Igamberdiev and Hill 2004; Reggiani et al. 1985; Stoimenova et al. 2007). Under low O_2_ and acidic conditions, the transcript level and activity of NR, which catalyzes the first step of NO_3_^−^ reduction to NO_2_^−^ in both pathways, are increased in some terrestrial species (Lager et al. 2010). The NO_3_^−^ reduction through the catabolic reduction pathway requires a large amount of NAD(P)H. In the NO_2_^−^-driven ATP synthesis cycle, about 2.5 mol NADH per 1 mol NO_3_^−^ is consumed. Thus, the flux to glycolytic fermentation decreases as a result of competition for NADH oxidation (Fan et al. 1988; Sowa et al. 1998).

In maize and barley roots, an increase in the NR activity under anaerobic conditions in the presence of NO_3_^−^ was observed to be accompanied by a decrease in the ethanol accumulation (Botrel and Kaiser 1997; Fan et al. 1988). In contrast, in roots of rice and *Carex* species (*C. pseudocyperus* L. and *C. sylvatica* Huds.) under anaerobic conditions, exogenous NO_3_^−^ stimulated anaerobic respiration (glycolytic fermentation) due to an accelerated glycolytic flux. This stimulation results from a more effective NADH reoxidation capacity by both NO_3_^−^ reduction and fermentation compared with only fermentation (Müller et al. 1994; Reggiani et al. 1985, 1993). Moreover, high capacity to use NO_2_^−^ as an electron acceptor strongly contributes to continuous ATP production in roots of rice and barley under anoxic stress (Stoimenova et al. 2007). This is because the NO_2_^−^-driven ATP synthesis cycle is activated by the addition of NO_3_^−^ under anoxic conditions (Fig. 3). These indicate that, under O_2_-deficient conditions, NO_3_^−^ has a favorable effect on the energy metabolism in roots of terrestrial as well as wetland plants.

The balance of the oxidation capacity of reducing equivalents (NADH) between the fermentation and the catabolic NO_3_^−^-reduction pathways may be different among species (Fig. 3). The protective effects of NO_3_^−^ utilization in rice shoots have been confirmed by analyzing their mitochondria using electron microscopy. In this study by Vartapetian et al. (2003), the marked destructive changes in the coleoptile mitochondria ultrastructure (membrane destruction, cristae disappearance, and pale matrix) were delayed until 48 h after the onset of anaerobic incubation in the presence of exogenous NO_3_^−^. In rice plants, NO_3_^−^ is reduced through the assimilative NO_3_^−^-reduction pathway in their shoots because they show high activities and transcript levels of NR and GS when they are grown in both NO_3_^−^ and NH_4_^+^ conditions (Yun et al. 2008). In contrast, under O_2_-deficient conditions, NO_3_^−^ is reduced to NO_2_^−^ by the catabolic NO_3_^−^-reduction pathway in their roots. These reports imply that the capacities of the two NO_3_^−^-reduction pathways, the assimilative and catabolic pathways, vary in the different tissues of a plant under anaerobic condition. Both pathways can contribute to hypoxic-stress tolerance through favorable effects on energy metabolism and cytoplasmic pH stabilization (Fig. 3).

#### NO_2_^−^-driven ATP synthesis in plants under O_2_-deficient conditions

Under O_2_ deficient conditions, exogeneous NO_3_^−^ is reduced to NO_2_^−^ by hypoxia-induced NR (Lager et al. 2010). When the O_2_ level falls below the saturation level of COX, mETC utilizes NO_2_^−^ as the electron acceptor instead of O_2_ for the maintenance of ATP synthesis and O_2_ concentration in cells (Gupta et al. 2005; Planchet et al. 2005) (Fig. 2). The rates of this NO_2_^−^-driven anaerobic ATP synthesis are of the same order as those of glycolytic ATP production during hypoxia, and about 3–5% of the aerobic mitochondrial ATP synthesis (Stoimenova et al. 2007). As NO produced by this NO_2_^−^-driven ATP synthesis is immediately converted to NO_3_^−^ through the hypoxia-induced Class 1 hemoglobin (Class 1 Hb), mETC components including COX are not damaged (Gupta and Igamberdiev 2011; Nie et al. 2006; Taylor et al. 1994) (Fig. 2). The expression of Class 1 Hb gene is triggered by a disruption of ATP synthesis and by Ca^2+^ release under O_2_-deficient conditions (Nie et al. 2006). Class 1 Hb has an extremely high affinity to O_2_, and its oxidized form, oxyHb, can oxygenate NO to NO_3_^−^ even at extremely low O_2_ concentrations (Trevaskis et al. 1997) (Fig. 2). In such a case, homeostasis of O_2_ and ROS is maintained because NO can tightly control respiration via inhibiting COX, which leads to an increase in the internal O_2_ levels under hypoxic conditions (Gupta et al. 2014). Thereafter, NO_3_^−^ is reduced to NO_2_^−^ by hypoxia-induced NR and recycled by the operation of this Hb/NO cycle (Gupta and Igamberdiev 2011; Igamberdiev and Hill 2004; Igamberdiev and Kleczkowski 2011). In the reaction when NO is converted to NO_3_^−^, the heme iron of Hb is oxidized to its ferric form, methemoglobin (metHb) (Fig. 2). To maintain the Hb/NO cycling, Class 1 Hb is regenerated from metHb by ascorbate (Fig. 2). The oxidized form of ascorbate, monodehydroascorbate, is reduced by monodehydroascorbate reductase (MDHAR) along with the oxidation of NAD(P)H (Igamberdiev et al. 2006; Loreti et al. 2005) (Fig. 2). High ascorbate level and induction of MDHAR are observed under hypoxia (Igamberdiev et al. 2006). Besides playing a role in the above cycle, ascorbate also has an important role in the detoxification of ROS and RNS such as H_2_O_2_, ONOO^−^, and O_2_^−^. In the NO scavenging process under hypoxia, ascorbate reduces NO to N_2_O in the mitochondrial matrix while monodehydroascorbate produced from ascorbate oxidizes ONOO^−^ to NO, and thus resupplies NO back to the cell (Alegria et al. 2004; Igamberdiev and Hill 2008) (Fig. 2). The Hb/NO cycling is influenced by the cytosolic NO_2_^−^ accumulation via high NR activation. This NR activation is induced by the decrease in ATP during hypoxia/anoxia, but inhibited by low NO_3_^−^ concentrations (Gupta et al. 2011; Planchet et al. 2005; Rockel et al. 2002; Stöhr and Mäck 2001). Thus, it seems that NO_2_^−^-driven ATP production may be an important strategy for hypoxia-tolerance in plants with high NR potential.

The turnover of NO and maintenance of the cellular redox and energy levels are strong evidence for NO_2_^−^-driven ATP production in some terrestrial plants, such as maize, alfalfa, and barley growing on NO_3_^−^ dominant soils under low O_2_ stress (Dordas et al. 2003; Igamberdiev and Hill 2004; Igamberdiev et al. 2006; Sowa et al. 1998). Moreover, NO_2_^−^-driven ATP production was also reported in rice plants that typically prefer NH_4_^+^ when exogenously supplied with NO_2_^−^ and NO_3_^−^ under anoxic conditions (Ohwaki et al. 2005; Stoimenova et al. 2007). The rate of anaerobic NO_2_^−^-driven mitochondrial ATP synthesis in rice was reported to be 25% of their total ATP turnover rate compared to that of 11.5% in barley during hypoxia (Stoimenova et al. 2007). These values were calculated based on the estimations that mitochondrial proteins represent 7% of the total proteins in heterotrophic plant cells (Douce 1985) and the rate of ATP turnover is 70 nmol min^−1^ mg^−1^ mitochondrial protein (Neuburger et al. 1996). This rate could be much higher in rice, 35% of the total ATP turnover rate, because the mitochondrial proteins of rice could comprise as much as 10% of the total proteins (Stoimenova et al. 2007). The ATP production per anaerobic mitochondrial NAD(P)H oxidation of rice is also higher than that of barley (Stoimenova et al. 2007). Thus, species that possess high potential of NO_2_^−^-driven ATP production system and contain abundant mitochondrial proteins such as rice plants, can increase their ATP production per anaerobic mitochondrial NAD(P)H oxidation when they utilize NO_3_^−^ as the N source. So far, the contribution of NO_2_^−^-driven ATP production system in wild wetland plants has been unnoticed because these plants prefer NH_4_^+^ in their habitats as nitrification is restricted by stagnant water. However, this system could become a crucial strategy in hypoxia-tolerant wild wetland species with a high ATP turnover rate, when NO_3_^−^ is available in their rhizosphere.

#### Differences in effects of NH_4_^+^ on respiration between terrestrial and wetland plants

The energy cost for NH_4_^+^ assimilation is lower than that for NO_3_^−^ (Bloom et al. 1992). However, many terrestrial plants need to assimilate NH_4_^+^ immediately after their absorption in the roots to avoid the toxicity symptoms associated with NH_4_^+^ as the sole N source (Britto and Kronzucker 2002). Concentrated NH_4_^+^ often increases the respiration rate (NH_4_^+^-dependent respiratory increase, ARI) in shoots, roots, and whole plants (Britto et al. 2001; Escobar et al. 2006; Hachiya et al. 2010). Thus, NH_4_^+^ utilization may lead to further O_2_ deficiency through ARI in many terrestrial plants when the N source is limited to only NH_4_^+^ by rhizosphere environmental changes such as submergence. In shoots and roots of terrestrial plants, ARI that is induced by an increase in NH_4_^+^ concentration in the external media (Britto et al. 2001) increases the ATP content and ATP/ADP ratio by inducing the phosphorylating components of mETC such as complex I, III, and IV (COX) (Curi et al. 2003; Hachiya et al. 2010; Welchen et al. 2002). However, these increases in respiratory ATP production are not related to an increase in useful energy demands such as growth. One of the main causes of ARI has been suggested to be an increase in the inward/outward flux of NH_4_^+^ across the plasma membrane, called “futile NH_4_^+^ cycling (FAC)” (Britto and Kronzucker 2002; Britto et al. 2001; Hachiya et al. 2010). As NH_4_^+^ uptake via the NH_4_^+^ transporter (AMT) is accompanied by proton extrusion from the plasma membrane H^+^-ATPase to maintain the cytosolic charge balance (Britto and Kronzucker 2002), the increased FAC under conditions of NH_4_^+^ as the sole N source would require more respiratory ATP (Britto et al. 2001). Consequently, ARI would occur to meet the increase in ATP demand related to increased FAC, when the plants are grown under high concentration of NH_4_^+^. Indeed, in some NH_4_^+^-intolerant terrestrial species such as maize and barley, the H^+^-ATPase activity is high when they are grown under conditions of NH_4_^+^ as the sole N source (Britto et al. 2001; Nielsen and Schjoerring 1998; Schubert and Yan 1997). In contrast, in the roots of NH_4_^+^-tolerant rice, ARI is not observed (Britto et al. 2001), and the activity of H^+^-ATPase is independent of the N source (Zhu et al. 2009). Moreover, the experiments in which NH_4_^+^ metabolism and growth rate are analyzed in rice plants have reported that the decrease in energy cost for FAC does not correlate with the optimized growth (Balkos et al. 2010). This low FAC in rice plants may reflect that they have evolved to be NH_4_^+^ tolerant without any energy cost to maintain the NH_4_^+^ balance across the plasma membrane (Karasawa et al. 1994; Kronzucker et al. 1999, 2001).

ARI is also explained by another hypothesis in which it occurs in relation to the dissipation of excess reducing equivalents in mETC. The NO_3_^−^ assimilation process competes with mETC for the reducing equivalents. The shift of an available N source from NO_3_^−^ to NH_4_^+^ increases the reducing equivalents that are not consumed through NO_3_^−^ assimilation and are thus available to be consumed by mETC, thereby increasing the O_2_ uptake rate (Bloom et al. 1992; Escobar et al. 2006). In particular, under low O_2_ stress conditions where COX is saturated with reducing equivalents, there is a possibility that the non-phosphorylating AOX and NDs in mETC can consume the excessive reducing equivalents without being limited by adenylate control (Escobar et al. 2006; Vanlerberghe et al. 2009). In fact, AOX capacity in terrestrial plants such as Arabidopsis, pea, and spinach increases when they are transferred from NO_3_^−^ to NH_4_^+^ conditions (Escobar et al. 2006; Frechilla et al. 2002; Lasa et al. 2002). NDB2, which is a major isoform of ND_ex_, is also induced in shoots and roots of Arabidopsis under NO_3_^−^-depleted conditions (Wang et al. 2004; Watanabe et al. 2010). Although these responses have an important role in the dissipation of excessive reducing equivalents under the low O_2_ stress conditions, ARI itself would lead to further strict anoxic threat for NO_3_^−^-preferring terrestrial plants under O_2_-deficient condition.

### Two contrasting adaptive strategies in flood-tolerant plants: the low oxygen escape strategy versus the low oxygen quiescence strategy

**Fig. 4:**
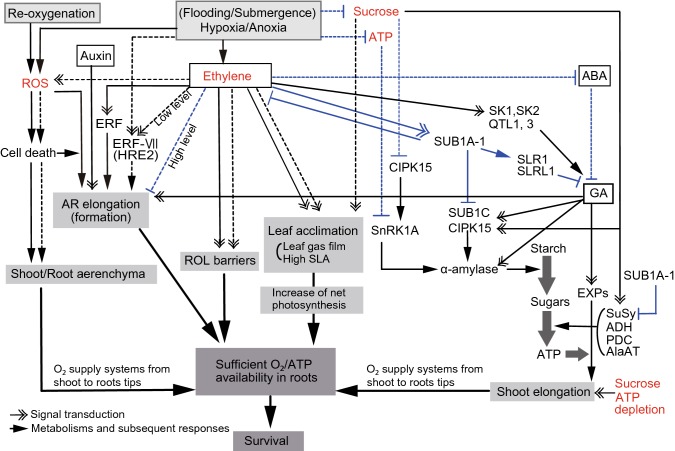
Characteristics of low O_2_ escape strategy (LOES) and low O_2_ quiescence strategy (LOQS) to hypoxia/anoxia caused by flooding/submergence in wetland and terrestrial plants. Black solid and dashed lines are the networks of LOES (aerenchyma formation, shoot elongation, radial O_2_ loss (ROL) barriers, and leaf acclimation) in wetland and terrestrial plants, respectively, while blue dashed lines indicate responses to suppress LOES in both plants; blue solid lines indicate the submerged regulatory network of LOQS in rice (wetland species). Four key factors, ROS accumulation, ethylene content, ATP depletion, and sucrose reserve decrease, involve the LOES and LOQS networks are shown in red letters. ROS production in hypoxic and anoxic stresses causes programmed cell death (PCD) in both plant types and involves the mechanisms of adventitious roots (ARs) emergence and aerenchyma formation. AR elongation in Arabidopsis (terrestrial plant) is promoted by the hypoxia signal and its formation is mediated by hypoxia-responsive HRE2, which is one of the group VII ethylene response transcription factors (ERFVIIs). High ethylene level inhibits the AR formation in Arabidopsis under hypoxic condition, although ARs are formed at low ethylene level. In contrast, in rice plants, ethylene has promotive effects on the AR formation and elongation. The contrasting regulation by ethylene on ARs may reflect different adaptive strategies in the flood-tolerant rice plants compared to the flooding-intolerant terrestrial species such as Arabidopsis. Leaf acclimation such as high specific leaf area (SLA), reoriented chloroplasts along with cell wall in leaf epidermis, thin cuticles and cell walls, development of dissected leaves underwater, and the maintenance of gas films can increase the net photosynthesis by decreasing the diffusion resistance for CO_2_. The leaf plasticity could also result from the accumulation of ethylene and a decrease in CO_2_ levels. Flooding/submergence causes ethylene accumulation, which triggers gibberellin (GA)-promoted cell elongation through the expansins (EXPs). In deep-water rice with LOES, ethylene promotes the induction of SNORKELs (*SKs*, *SK1*, and *SK2*) and GA elevation and the internodes of the shoots elongate rapidly to come out of the water surface. In the deep submergence lines of rice with LOQS, ethylene activates the submergence 1A-1 (*SUB1A*-*1*) promoting an increase in SLENDER RICE 1 (SLR1) and SLENDER RICE-Like 1 (SLRL1) transcription factors, which inhibit GA-mediated activation of gene expressions. This LOQS characteristic of rice can limit carbohydrate consumption by inhibiting shoot growth. Wetland plants develop shoot and root aerenchyma, ROL barriers, and elongated shoots elongation and these characteristics of LOES act synergistically with each other in enhancing the stability of O_2_ and ATP availability in roots where nitrogen (N) uptake and active N assimilation take place. Abbreviations are as follows: ABA, abscisic acid; ADH, alcohol dehydrogenase; AlaAT, alanine aminotransferase; CIPK15, calcineurin B-like interacting protein kinase 15; HRE2, hypoxia-responsive ERF 2; PDC, pyruvate decarboxylase; *QTL1* and *3*, quantitative trait loci on chromosomes 1 and 3; SnRK1A, sucrose non-fermenting receptor kinase 1A; SuSy, sucrose synthase; *SUB1A*-*1*, submergence 1A-1

Flood tolerant plants that can survive at O_2_ deficiency or light-limited submergence conditions are characterized by two survival strategies. One of them is the low O_2_ escape strategy (LOES), and the other is the low O_2_ quiescence strategy (LOQS) (Fig. 4). Plants with LOES phenotypes show upward bending of leaves (hyponasty) that can enhance shoot elongation, formation of interconnected air-filled voids (aerenchyma), induction of barriers to radial O_2_ loss (ROL) in roots, development of adventitious roots (ARs), formation of gas films on leaf surfaces, modification in leaf anatomy, and pressurized gas flow through porous tissues under O_2_-deficient conditions. All of these characteristics are not necessarily found in one species (Blom 1999; Evans 2004; Jackson and Armstrong 1999; Ridge 1987; Sauter 2013) (Fig. 4). The elongation of aerial organs and the formation of aerenchyma and ARs are all ethylene dependent. The former trait is also controlled by a hormonal network, which includes ABA and GA, and the latter trait by ROS (Voesenek and Bailey-Serres 2015) (Fig. 4). In contrast, plants with LOQS phenotypes cease their growth and save their carbohydrate reserves under O_2_-deficient conditions.

#### Molecular mechanisms of LOES and LOQS

In rice varieties, both LOES and LOQS are found to counteract flooding stress. *SNORKEL1* (*SK1*) and *SNORKEL2* (*SK2*) of deep-water rice varieties (LOES type) are involved in the rapid internode elongation and escape of leaves near the water surface (Hattori et al. 2009), whereas *submergence 1A*-*1* (*SUB1A*-*1*) in lowland varieties of rice (*indica*) (LOQS type) limits elongation, growth, and carbohydrate consumption (Fukao and Bailey-Serres 2008; Fukao et al. 2006) (Fig. 3). The key regulatory genes in both strategic responses are the ethylene-responsive TFs of the subfamily group VII (ERF-VII); the TFs act downstream of ethylene and modulate GA-mediated shoot growth (Bailey-Serres and Voesenek 2010; Voesenek and Bailey-Serres 2015). Deepwater varieties of rice (LOES type) can escape from adverse partially submerged deep-water conditions through *SK1* and *SK2* genes (*SKs*) that trigger rapid internode elongation at a rate of 25 cm day^−1^ (Colmer et al. 2014; Hattori et al. 2011) (Fig. 4). In contrast, these genes are absent in shallow water varieties including all *japonica* varieties (LOQS type). Moreover, two additional uncharacterized loci on chromosomes 1 and 3 (*QTL1* and *3*) are needed along with *SKs* for the full deep-water escape response (LOES type) (Ayano et al. 2014). Shoot and internode elongations in submerged deep-water varieties of rice are promoted by cell expansion and division, which are positively regulated by ethylene and GA (GA_1_ and GA_4_). These hormones enable expansins (EXPs) and α-amylase to drive cell elongation and starch degradation by *SKs* and *QTL1* and *3* (Choi et al. 2004; Rzewuski and Sauter 2002; Sauter et al. 2002; van der Knaap et al. 2000) (Fig. 4).

In contrast, the *indica* varieties possessing *SUB1A*-*1* (LOQS type) decrease their metabolic activities and constrain their growth to save energy consumption under shortly prolonged submergence conditions (up to only a few weeks) (Fig. 4). The *indica* and *japonica* varieties lacking the *SUB1A* gene or *SUB1A*-*1* allele cannot cease their metabolic activities (Fukao et al. 2006; Xu et al. 2006). *Submergence 1* (*SUB1*) locus of rice consists of three genes, *SUB1A*, *SUB1B,* and *SUB1C*. The expression of *SUB1A*-*1* alone is sufficient to provide flood tolerance, but it exists only in flood-tolerant varieties with LOQS traits. *SUB1C* is present in all varieties, and responds to GA and positively regulates the expression of several EXPs (Fukao and Bailey-Serres 2008; Hattori et al. 2011; Xu et al. 2011). *SUB1B* is ERF similar to *SUB1C* (Bailey-Serres et al. 2010). The submergence-intolerant *japonica* cultivar Nipponbare has both *SUB1B* and *SUB1C,* but lacks *SUB1A*. *SUB1A*-*1* inhibits shoot elongation by maintaining the levels of TFs, SLENDER RICE 1 (SLR1) and SLENDER RICE-Like 1 (SLRL1), to counterbalance the GA responsiveness and regulate the *SUB1C* mRNA level negatively (Bailey-Serres and Voesenek 2008; Fukao et al. 2006) (Fig. 4). Furthermore, *SUB1A*-*1* negatively regulates the submergence-induced synthesis of ethylene, mRNA expression of cell-wall-loosening EXP, starch and sucrose degradation (Fukao et al. 2006), and chlorophyll degradation through zinc-finger TF encoded by DELAY OF THE ONSET OF SENESCENCE (Fukao et al. 2012; Winkel et al. 2014). The elongation processes through *SUB1C* require a large amount of energy during shoot submergence because elongation of aerial organs is accompanied with the rapid and efficient translocation of photosynthates and reserved carbohydrates and amino acids (Ayano et al. 2014; Hattori et al. 2011; Kende et al. 1998; Sauter 2000). In contrast, LOQS varieties with *SUB1A*-*1* can decrease their energy utilization until the water level decreases and normoxic conditions are restored, thereby they resume growth with preserved energy under subsequently normoxic conditions (Ayano et al. 2014; Barding et al. 2012, 2013; Fukao and Bailey-Serres 2008; Hattori et al. 2011; Kende et al. 1998; Nagai et al. 2010; Sauter 2000). The rice varieties with *SUB1A*-*1* can restrict the rate and extent of starch hydrolysis and accumulate lower concentrations of ethanol, lactate, and amino acids than the varieties without *SUB1A* (Barding et al. 2012, 2013). It has been assumed that repeated elongation of aerial tissues in every short-term submersion may damage the growth by serious re-oxidation and water loss in the LOQS phenotypes (Hattori et al. 2011; Nagai et al. 2010). The varieties with *SUB1* can manage ROS accumulation and leaf water loss during recovery from submergence conditions to a minimum extent. This is because they have higher levels of mRNA associated with the repression of ROS accumulation during the recovery phase (Fukao et al. 2011; Mustroph et al. 2010).

In wild wetland plants such as *Rumex palustris*, *R. acetosa. Sagittaria trifolia* and *Lotus tenuis*, it has been suggested that there are networks in conserved flooding response that relate to growth and stress-induced catabolism of carbohydrates for the efficient ATP production. However, studies on ERF-VII TFs (*SKs* and *SUB1*) are required in wild wetland species that experience long-term flooding (Kim et al. 2000; Manzur et al. 2009; Ookawara et al. 2005; Vreeburg et al. 2005). In these plants, there are considerable genetic variations between and within species in the ethylene-induced elongation capacity under submergence conditions. It is noteworthy that the wild species *R. palustris* displays submergence escape by ethylene-driven shoot elongation (LOES type) (Benschop et al. 2005), and *R. acetosa* invokes quiescence owing to a lack of ABA down-regulation, GA up-regulation, and increased EXP expression (LOQS type), although these two species are closely related to each other (Benschop et al. 2005; Chen et al. 2009; van Veen et al. 2013; Vriezen et al. 2000). In *R. palustris*, it seems that the elements downstream of ethylene and upstream of ABA and GA can switch on this elongation cascade (Benschop et al. 2005; Chen et al. 2009; van Veen et al. 2013). Moreover, *R. palustris* exposed to dark under submergence conditions can convert their strategy from escape to quiescence for survival. This strategy is achieved by the pretreatment using ethylene, in which LOQS ability is promoted (van Veen et al. 2013). This strategy conversion in *R. palustris* relates to the light-signaling genes that regulate the enhancement of shoot elongation (van Veen et al. 2013), and this observation demonstrates the similarity of growth control between shade avoidance and underwater elongation. Another wetland species, *L. tenuis,* also elongates upon partial submergence but arrests its growth upon complete submergence. It switches from LOES to LOQS due to elevation of shoot porosity and limited consumption of soluble carbohydrates in shoots and roots (Manzur et al. 2009). Both antithetical LOES and LOQS strategies exist within a single species and are not mutually exclusive. These may be combined by the threshold of O_2_ level or energy deficiency. In this regard, Voesenek and Bailey-Serres (2015) indicate that three key factors, an increase of cellular ethylene content, depletion of ATP, and consumption of readily available sucrose in the submergence network, can contribute to increased induction and regulation of shoot elongation. The level of reserved carbohydrates for ATP production seems to strongly affect the strategies for sustainable and facilitative survival in various natural flooding environments (Fig. 4). Especially the LOES type may be required for the high photosynthetic capacity and translocation activity of photosynthates and reserves under submergence conditions. Therefore, the interplay among hormones (ethylene, ABA, and GA), O_2_ availability, and specific metabolites (ATP, sugars, and pyruvate) needs further clarification for understanding the network balancing growth and quiescence.

#### Avoidance strategies in LOES-type plants for improvement of O_2_ level within plant tissues

When plants are submerged by flooding, species with LOES phenotypes respond to O_2_ deficiency for improvement of cellular O_2_ level. Shoot elongation, formation of interconnected air-filled voids (aerenchyma), pressurized gas flow through the aerencyma and leaf acclimations for the decrease of the diffusion resistance to air can function to improve cellular O_2_ levels. In the roots, developed aerenchyma, formation of the ROL barrier from the roots surface and development of ARs can enhance the longitudinal O_2_ diffusion in root tips with the most active cells.

##### Aerenchyma

Aerenchyma can decrease the gas diffusion resistance from the atmospheric tissues to the O_2_-deficient tissues. The formation of aerenchyma and enhancement of the gas transport ability are essential strategies in LOES (Fig. 4). O_2_ produced during photosynthesis or taken up by the aerobic shoots diffuses inside the aerenchyma connecting the shoots and the roots; this O_2_ diffusion supports respiration in O_2_-deficient underwater organs (Fig. 4). Aerenchyma can be formed by different processes such as schizogeny and lysigeny (Drew et al. 2000; Evans 2004; Seago et al. 2005). These processes often appear simultaneously at different organs in one individual plant (Steffens et al. 2011). Although little is known about the process of schizogenous aerenchyma formation (Evans 2004), it has been hypothesized that the causal protein, NOP1, regulates the schizogenous formation of air chambers via a membrane-localized receptor-like kinase signaling pathway resulting in ubiquitylation and degradation of target proteins (Ishizaki et al. 2013). In contrast, the lysigenous formation of air chambers via programmed cell death (PCD) requires ethylene, Ca^2+^, and ROS signaling, which ultimately breaks down the cell walls as observed in some species such as rice, Arabidopsis, maize, and wheat (Drew et al. 2000; Evans 2004). In roots of maize and deep-water rice, studies have reported that aerenchyma formation is associated with the accumulation of ROS and down-regulation of *METALLOTHIONEIN 2b* mRNA encoding a ROS scavenging protein (Rajhi et al. 2011; Steffens et al. 2011). Moreover, it seems that the Ca^2+^-dependent plasma membrane-localized respiratory burst oxidase homologs (*RBOHs*) influence the ROS sources in this process as they have been reported to promote apoplastic superoxide production to amplify ROS-mediated signaling in wheat and rice (Parlanti et al. 2011; Yamauchi et al. 2013b).

##### Pressurized flow-through system

In addition to the inward diffusion, a mechanism of gas transport in all wetland plants, some floating plants and macrophytes have a pressurized flow-through system in stems and rhizomes to aerate the O_2_-deficient underwater organs (roots and rhizome). This system enables the underwater organs to keep the O_2_ concentration to an ambient level for maintaining oxidative phosphorylation, thereby normalizing the ATP concentration (Colmer 2003; Sorrell and Hawes 2009) (Fig. 4). Pressurized flow-through is produced by the species-specific positive pressure capacity in shoot tissues and the resistance to the flow in the aerenchyma. Also, leaf-to-air gradients of temperature and humidity affect the pressurized flow (Brix et al. 1992; Colmer 2003). This flow mechanism also contributes to an outward diffusion of ethylene generated in roots and methane generated in soils (Colmer 2003; Laanbroek 2009). Thus, pressurized flow-through that facilitates effective gas flow through developed aerenchyma seems to provide a competitive advantage to large varieties of plants in deep-water habitats (Konnerup et al. 2011).

##### Leaf acclimation

In terrestrial plants, the net photosynthesis of submerged leaves often decreases significantly compared with that of aerial leaves due to an exponential decrease in light intensity with increasing depth and resistance to CO_2_ and O_2_ fluxes in submerged leaves (Colmer et al. 2011; Herrera 2013). In response to submergence, some terrestrial plants develop new acclimated leaves that are characterized by higher specific leaf area (SLA) that is a ratio of leaf area to leaf mass, reoriented chloroplasts along with cell walls in leaf epidermis, thin cuticles and cell walls, development of dissections, and maintenance of gas films (Colmer et al. 2011; Mommer et al. 2005b) (Fig. 4). All of these traits can decrease the diffusion resistance to CO_2_, thereby enabling the leaves to increase the net rate of CO_2_ assimilation and decrease the CO_2_ compensation point under water (Mommer et al. 2006; Pedersen et al. 2013; Winkel et al. 2014).

Wetland plants, such as *Rumex palustris*, *R. acetosa* and rice, show plastic acclimation of their morphological, anatomical, and biochemical traits of leaves to submergence (Mommer et al. 2005a, b; Pedersen et al. 2009, 2013; Winkel et al. 2014). *Ranunculus repens* constitutively dissect their leaves when they grow underwater (He et al. 1999). In *R. palustris*, the new acclimating leaves developed in underwater conditions can lead to a nearly 40-fold decrease in the diffusion resistance to CO_2_ (Mommer et al. 2005a). Rice plants show a large variation in these leaf traits among varieties. Submergence-tolerant landrace FR13A with LOES has higher net underwater photosynthesis, longer retention of the leaf gas film and longer persistence compared with a Sub1 variety, Swarna-Sub1 (Winkel et al. 2014; Xu et al. 2006). These traits of FR13A contribute to submergence tolerance because the persistence of gas film could potentially increase net photosynthesis and internal aeration during submergence. In contrast, in Swarana-Sub1, the duration of gas film retention is shorter than that in FR13A, although Swarana-Sub1 can maintain carbohydrate levels during submergence. Since both varieties have *SUB1A*, genetic determinants other than *SUB1A* contribute to gas film formation and underwater photosynthesis. Leaf acclimation ability to submergence may be related to flood tolerance in wetland plants with LOES. In contrast, these acclimations may not be related to flood tolerance in terrestrial plants (Mommer et al. 2007). The accumulated ethylene does not necessarily function as a signal for the flood-induced leaf acclimation in terrestrial plants under flooded conditions, but other signals associated with changed photosynthetic rates and/or decreased levels of carbohydrates may induce these leaf acclimations (Bailey-Serres and Voesenek 2008) (Fig. 4).

##### Barrier to radial O_2_ loss

The O_2_ supplied from the aboveground to underground organs diffuses to the anaerobic soil as radial O_2_ loss (ROL), which contributes to protecting the roots from toxic ions (Fe^2+^and Mn^2+^) and to the nitrification in NH_4_^+^-predominant and excessive-reduced soils by the oxidized layers around the roots. Moreover, wetland plants and some terrestrial plants form an impermeable barrier to ROL from the root basal zone to the apex (ROL barrier) by the deposition of suberin in the root exodermis (Fig. 4). The suberin layer is mainly composed of long-chain fatty acids. The ROL barrier acts synergistically to enhance the longitudinal O_2_ diffusion in the root tips with the most active cells and enables the development of aerobic rhizosphere around the root tips for root extension (Abiko et al. 2012; Armstrong and Beckett 1987; Colmer 2003; Sauter 2013) (Fig. 4). The ROL barrier is permanently formed in some wetland species, or temporarily induced by waterlogging in rice and wheat (Colmer 2003; Kotula et al. 2009; Malik et al. 2011) (Fig. 4). Molecular investigations in rice plants have clarified that the ROL barrier formation involves the up-regulation of genes including a hypodermal cell ABC transporter (*REDUCED CULM NUMBER1* [*RCN1*]/*OsABCG5*), which is proposed to export the long-chain fatty acids and/or their derivatives across the hypodermal plasma membrane into the apoplast to induce hypodermal suberization (Shiono et al. 2014). Indeed, the metabolite profile analysis in rice roots growing under barrier-forming stagnant conditions reveals that the concentrations of long-chain fatty acids and malate, which is a substrate for fatty acid biosynthesis, gradually increase from the root apex to the base (Kulichikhin et al. 2014).

##### Adventitious roots

Adventitious roots (ARs) are also associated with conferring developmental plasticity to plants under waterlogged condition. ARs with high porosities emerge from submerged stem nodes and hypocotyls to replace the existing and deteriorating primary root system in rice, *R. palustris*, *Solanum lycopersicum*, and *Larix laricina* (Calvo-Polanco et al. 2012; Dawood et al. 2014; Dawood et al. 2016; Eysholdt-Derzsó and Sauter 2019; Visser et al. 1996; Yang et al. 2018; Zhang et al. 2017) (Fig. 4). Some ARs develop chloroplasts and thus provide an additional source of O_2_ and carbohydrates (Rich et al. 2012) because ARs typically develop in well-aerated topsoil layers (Dawood et al. 2014; Eysholdt-Derzsó and Sauter 2019; Zhang et al. 2015). The terrestrial plant *Solanum dulcamara* can survive under flooding condition by replacing the original flood-sensitive root system with aerenchymatous ARs that are produced from pre-formed primordia on the stem. The AR outgrowth is involved with auxins, ABA, and jasmonic acid (Dawood et al. 2016; Vidoz et al. 2010; Yang et al. 2018). ABA is a negative regulator of AR outgrowth, but there is a highly tissue-specific response to decreased ABA levels. Auxins may be necessary for AR outgrowth because a disruption in the auxin signaling in AR primordia of *S. dulcamara* resulted in the abortion of AR outgrowth under complete submergence (Dawood et al. 2016) (Fig. 4). Moreover, the auxin pathways act together with decreased levels of ABA because the AR emergence in *S. dulcamara* was not sufficient when they were treated with auxin alone (Yang et al. 2018).

In Arabidopsis, low levels of ethylene and hypoxia signals mainly promote AR elongation due to the expression of the hypoxia-responsive *HRE2* which is one of the ERFVII TFs (Bailey-Serres et al. 2012; Eysholdt-Derzsó and Sauter 2019; Hess et al. 2011) (Fig. 4). However, high levels of ethylene inhibit the initial formation of ARs because high ethylene concentration can override the hypoxia signal (Eysholdt-Derzsó and Sauter 2019). Thus, the formation and elongation of ARs in Arabidopsis are controlled by ethylene in a dose-dependent manner (Fig. 4). Although low levels of ethylene in Arabidopsis may contribute to fast elongation of ARs immediately after exposure to hypoxia, they cannot exert hypoxia tolerance. This is because they do not form subsequent ARs when exposed to long-term severe anaerobic stress associated with high ethylene accumulation. In contrast to Arabidopsis, ethylene has promotive effects on the AR emergence and growth in several wetland species including rice (Lin and Sauter 2018). The contrasting regulation of ethylene on ARs may reflect the different adaptive strategies between the flood-tolerant and intolerant species (Fig. 4).

The roles of these phytohormones in the terrestrial species would be different from those in rice plants, especially in the AR emergence pathway. The emergence of the AR primordia in rice plants involves PCD in the overlying epidermal cells, which is mediated by ethylene-promoted ROS production (Steffens et al. 2013) (Fig. 4). The developmental process of ARs involves the up-regulation of the plasma membrane *RBOHs* (Steffens et al. 2012), and the decrease in *METALLOTHIONEIN 2b*, which regulates the ROS amelioration for nodal AR emergence (Voesenek and Bailey-Serres 2015). The location of this PCD is determined by the force exerted by the outgrowing meristems, and at the same place, epidermal weakening for emergence of AR primordium can be elicited by the essential degradation of pericycle and epidermal cells by cell wall-modifying proteins such as EXPs, subtilisin-like proteases, pectate lyases, and endo-β-1,4-glucanases (Cho and Kende 1997; Kimpara et al. 2008; Laskowski et al. 2006; Steffens et al. 2012; Yamauchi et al. 2013a).

##### Diverse strategies for avoidance from O_2_ deficiency

In rice and some wetland species, ROL barrier and leaf acclimation improve their underwater photosynthesis, root aeration, and growth (Colmer and Pedersen 2008; Pedersen et al. 2009; Winkel et al. 2013, 2014) (Fig. 4). These characteristics act synergistically with each other to enhance flood tolerance in wetland species (Fig. 4). In contrast, acclimated leaves with morphological modifications and ARs cannot provide flooding tolerance to terrestrial plants because O_2_ cannot be transported to the underwater organs of terrestrial plants (Fig. 4). As roots consume large amounts of O_2_ for nutrient uptake, they often suffer from O_2_ deficiency. The internal O_2_ pressure in roots is much lower than that in shoots, especially at night when photosynthetic O_2_ production ceases (Pedersen et al. 2006). Therefore, effective aerenchyma is required to enable the roots to sustain the oxidative phosphorylation leading to normal ATP production and growth (Visser and Pierik 2007). However, despite the benefits of aerenchyma under flooded conditions, it is not constitutive in all plants. In this regard, Striker et al. (2007) described the significant trade-off between root porosity and mechanical strength. Hu et al. (2013) also confirmed that aerenchyma inhibits radial nutrient transport in maize roots. Therefore, it could be assumed that many wetland plants can maintain an optimal balance between their growth and low O_2_ tolerance in their roots, and this balance is significantly different from that in the terrestrial plants.

#### Traits of root N use and O_2_ uptake in LOES-type wetland plants under O_2_-deficient conditions

**Fig. 5:**
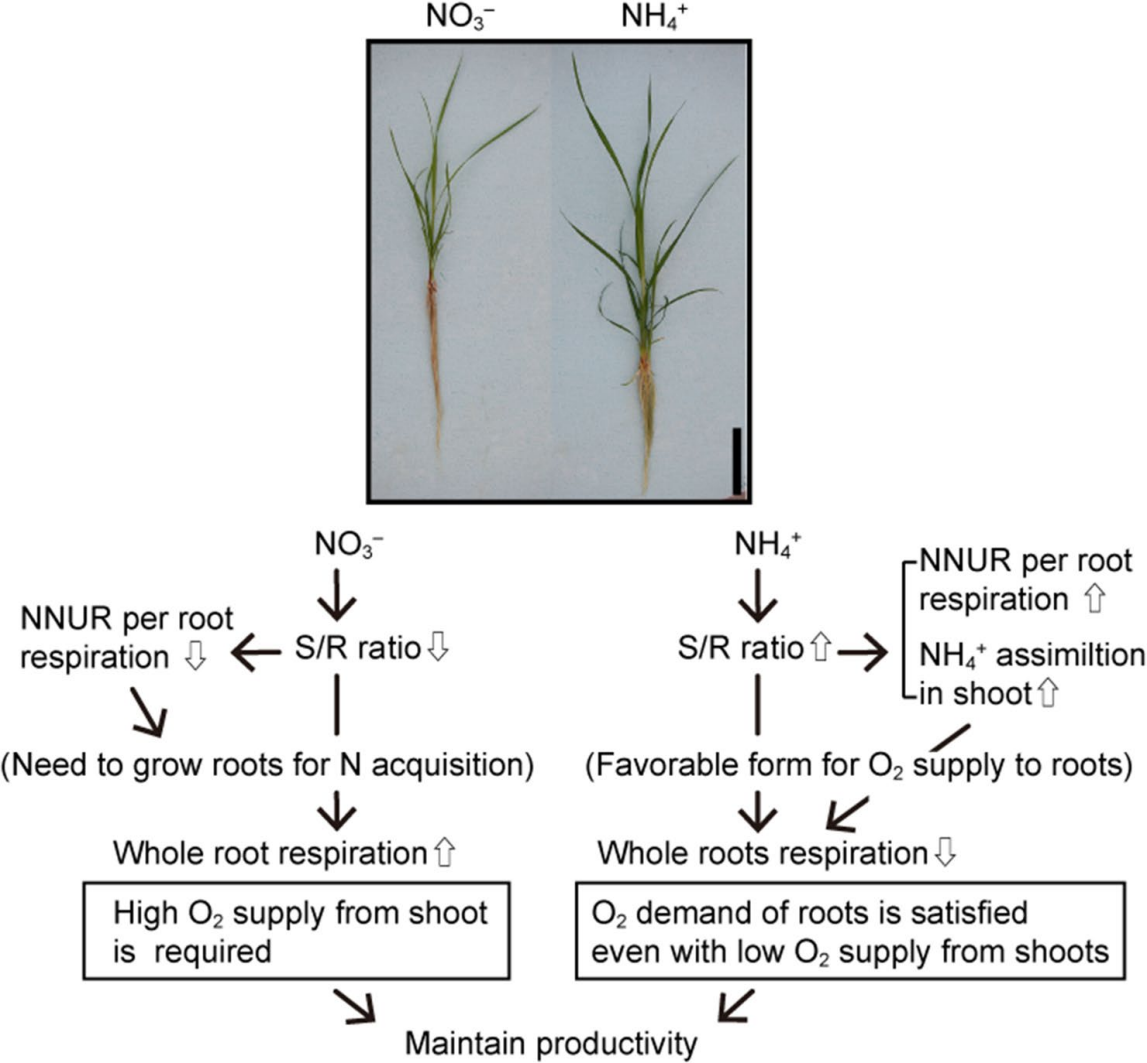
Effects of NO_3_^−^ (left) and NH_4_^+^ (right) utilization as the sole nitrogen (N) source on root respiration and N acquisition in wetland plants. The downward (⇩) and upward (⇧) arrows indicate the decreasing and increasing responses, respectively. NO_3_^−^ utilization results in a low root to shoot weight (S/R) ratio, which is unfavorable for O_2_ supply. As the N uptake rate per root weight (NNUR) per root respiration rate decreases when the wetland plants utilize NO_3_^−^, they develop the roots for N acquisition, consequently increasing the respiration of the whole roots. Therefore, NO_3_^−^ utilization requires high O_2_ supply to maintain productivity. In contrast, NH_4_^+^ utilization results in a high S/R ratio, which is favorable for O_2_ supply, and high NNUR per root respiration. Moreover, when NH_4_^+^ concentrations increase, the wetland plants may assimilate NH_4_^+^ in their shoots instead of their roots. These traits contribute to a decrease in the respiration of the whole root, and thus wetland plants can ensure NH_4_^+^ utilization even under low O_2_ supply. Photograph of *Carex lyngbyei* grown in 200 µM NO_3_^−^ and NH_4_^+^ treatments under hypoxic hydroponic culture for 1 month. Bar 5 cm

Differences in the rhizosphere N conditions such as sole or mixture of NO_3_^−^ and NH_4_^+^ generally change root density, extension, and whole weight. These changes alter the rhizosphere pH and redox potential, which regulate the root cell proliferation and mechanical properties (Bloom et al. 2003; Brix et al. 2002; Marschner and Römheld 1983). Because NH_4_^+^ dominates as the inorganic N source under anaerobic soil conditions due to the limitation of nitrification, NH_4_^+^-tolerant wetland species ameliorate the toxic effect of excess NH_4_^+^ by exhibiting high GS activity for the quick assimilation of NH_4_^+^ compared with that of NH_4_^+^-intolerant terrestrial plants (Balkos et al. 2010; Britto and Kronzucker 2002). As rice plants have isoenzymes of the cytosolic GS1 gene family (*OsGLN1;1* and *OsGLN1;2*) that can be classified into high-affinity subtypes with relatively high V_max_, these GSs facilitate active NH_4_^+^ assimilation in their roots (Ishiyama et al. 2004). Studies on NH_4_^+^ metabolism in rice, maize, and tomato plants have reported that rice plants have much higher GS activity than the other species, and the GS activity increased more in the shoot tissues than that in the roots with the increase in NH_4_^+^ (Magalhaes and Huber 1991). This indicates that the GS activity is a key factor in the detoxification and assimilation of NH_4_^+^ in shoots of plant species with efficient NH_4_^+^ utilization (Magalhaes and Huber 1991). The ability to rapidly assimilate NH_4_^+^ not only in roots but also in shoots can also function as an N acquisition strategy in the wetland species growing under O_2_-deficient conditions. This is because the up-regulated N assimilation in shoots can lead to decrease in the demand for N assimilation in the roots, thereby decreasing root respiration and avoiding O_2_ deficiency in roots (Fig. 5). When wetland wild grass and *Carex* species (*C. lyngbyei, C. lasiocarpa* var. *occultans*, and *C. middendorffii*) are grown in the sole NH_4_^+^ treatment under low O_2_ condition, they exhibit a smaller root to shoot weight ratio (i.e., high S/R ratio) and increased net N uptake rate per unit root weight (NNUR) compared to the sole NO_3_^−^ treatment. This high S/R ratio can lead to the decrease in the whole-root O_2_ consumption in the sole NH_4_^+^ treatment (Nakamura and Nakamura 2016; Nakamura et al. 2010, 2013) (Fig. 5). The decreased root growth (high S/R ratio) with sole NH_4_^+^ treatment is more prominent in species with weak O_2_-supply system with only diffusion than in species with strong O_2_-supply system with pressurized gas flow (Nakamura et al. 2013) (Fig. 5). Although the high NNUR causes high root respiration rate per unit root weight in species with low O_2_-supply system, the whole root respiration rate per shoot weight is similar between sole NH_4_^+^ and NO_3_^−^ conditions due to the compensation for high O_2_ uptake in roots by their high S/R ratio under sole NH_4_^+^ condition (Fig. 5). Thus, it seems that wetland plants primarily employ the NH_4_^+^ utilization strategy for N-acquisition, which enables them to acquire sufficient N for their growth and to minimize and regulate the whole-root O_2_ consumption depending on the O_2_ supply from shoots to roots (Fig. 5).

Even in anaerobic soil dominated by NH_4_^+^, small amounts of NO_3_^−^ are produced by oxidization in the soil due to the O_2_ flux to the rhizosphere by species with active ROL (Brix et al. 2002; Kirk and Kronzucker 2005). When NH_4_^+^-preferring species such as rice are grown under conditions where both NH_4_^+^ and NO_3_^−^ are supplied, they show improved productivity and increase in the net N acquisition and ATP production (called “the synergistic effect of NH_4_^+^ and NO_3_^−^”) compared with only sole NH_4_^+^ conditions (Kirk and Kronzucker 2005; Kronzucker et al. 1999; Li et al. 2006; Ying-Hua et al. 2006, 2007). Some studies have also reported the underlying mechanisms by which the addition of NO_3_^−^ to an NH_4_^+^ containing soil increased the *V*_max_ value of NH_4_^+^ uptake and plasma membrane potential due to an increase in the number of NH_4_^+^ transporters, leading to enhanced growth and N uptake in rice plants (Ying-Hua et al. 2006, 2007). Moreover, the synergistic effect of NH_4_^+^ and NO_3_^−^ in rice differs among varieties depending on the supply level of each inorganic N source and is genetically controlled (Ancheng et al. 1993; Ying-Hua et al. 2007). The synergistic effect of inorganic N has been studied in wild wetland plants. The synergistic effect of NH_4_^+^ and NO_3_^−^ may be limited to species growing in habitats where nitrification occurs in their rhizosphere. Especially, the fast-growing species with high O_2_-supply system may display high ATP production and N acquisition under NH_4_^+^-dominant soil conditions due to this synergistic effect (Fig. 5).

The acquisition abilities of each inorganic N source in the soil are different among species with different O_2_-supplying abilities even when they exist in similar habitats. *Phragmites australis* and *Zizania latifolia* are observed in the same habitat, but the abilities of O_2_ supply are different. *P. australis* has a high ability of O_2_ supply by the convective gas flow system and high ability of NO_3_^−^ use owing to relatively high NR activity in roots under sole NO_3_^−^ and^−^ low O_2_ conditions. In contrast, *Z. latifolia* with only diffusion as the O_2_ supply system cannot survive under such conditions because of low activity of NR (Nakamura et al. 2013). Moreover, species with high ability of NO_3_^−^ use can utilize the NO_2_^−^-driven ATP production system under O_2_-deficient conditions, but species without the ability of NO_3_^−^ use can only utilize the fermentation system for ATP production under O_2_-deficient conditions (Fig. 5). Thus, the utilization ability of inorganic N depending on the O_2_ supplying capacity might be related not only to the N acquisition strategy but also to the anaerobic ATP production pathway under O_2_-deficient conditions. This suggests that low O_2_ tolerance is characterized by the functional linkage between N utilization strategy and O_2_-supply capacity for the anaerobic energy conservation in wetland plants (Fig. 5). Stoimenova et al. (2007) have reported that rice plants with a low O_2_-supplying diffusion system can have the NO_2_^−^-driven ATP production system in the presence of exogenous NO_3_^−^ under anoxic condition, and this can strongly contribute to energy maintenance under anoxic conditions.

In general, NO production is elevated under low O_2_ conditions via the catabolic pathway from NO_2_^−^ (NO_2_^−^-dependent NO production) (Planchet et al. 2005; Rockel et al. 2002). In root cortex under hypoxic condition, NO is released from organelles when it is not scavenged by Class 1 Hb involving the NO_2_^−^-driven ATP synthesis cycle and other NO detoxification systems in the cytosol. NO increases ethylene that activates the signal transduction pathway involving phosphoinositides and Ca^2+^, and thereby aerenchyma formation is induced through PCD (Dordas et al. 2003; Drew et al. 2000; Voesenek and Bailey-Serres 2015; Yamauchi et al. 2013a). In transgenic alfalfa root cultures expressing the antisense barley Hb transcripts, the NO level was not changed, and cell breakdown and aerenchyma formation were induced when the cells were subjected to hypoxia (3% O_2_). In contrast, no cell breakdown was reported in the overexpressing Hb line under the same growth conditions (Dordas et al. 2003). Most wetland plants can develop aerenchyma in their shoots and roots, but the mechanisms of aerenchyma formation and their development level may differ among species depending on their NO_3_^−^ utilization ability and the NO levels. Further analyses are required to compare the effects of NO_3_^−^ utilization on the aerenchyma formation among species with different O_2_ supply capacities.

The formation of root cortical aerenchyma can also be induced by nutrient deficiency in the terrestrial species, maize (Hu et al. 2013). The formation of root cortical aerenchyma decreases the radial transport of nutrients by decreasing the living cortical tissue, which leads to a decrease in the maintenance requirements of living tissues of roots (Hu et al. 2013). Similarly, aerenchyma formation in wetland plants may contribute to not only avoid O_2_ deficiency in root tips but also decrease the respiratory energy required to maintain the living tissues under O_2_ limiting conditions. This saving of energy consumption by the aerenchyma formation may increase the allocation of the respiratory energy to other processes such as root growth and nutrient uptake. Some wetland plants with developed aerenchyma allocate their root respiratory ATP to maximize the N uptake instead of root maintenance and growth (Nakamura and Nakamura 2016). Such root responses in wetland plants could be their strategy for efficient O_2_ consumption and high N acquisition for adapting to O_2_ deficiency.

## Conclusion

Wetland species with hypoxia and anoxia tolerance can regulate their carbohydrate level to maintain the glycolytic flux and reduce ATP consumption under O_2_-deficient condition. At low O_2_, NAD(P)^+^ regeneration by ethanol fermentation, sucrose degradation through the energy-saving SuSy pathway, and amino acid metabolisms such as glutamate, GABA, and alanine are common in both wetland and terrestrial species. Gene expression of α-amylase in the aleurone layer and storage organs at the germination and initial growth stages are limited to wetland species. Moreover, an effective tolerant function in the wetland species for surviving long-term hypoxic and anoxic conditions is caused by maintaining glycolysis through the reversible reaction catalyzed by PPDK and PFK-PP_i_. In rice plant, cytosolic PPDK is more abundant in their roots than that in their shoots, and this may affect their adaptive response to frequent fluctuations in O_2_ concentration. In post-hypoxic/anoxic stress, the metabolic change from glycolysis to gluconeogenesis and to the TCA cycle contributes to normal metabolism during the recovery phase of re-oxygenation.

In wetland plants, the quick response of non-phosphorylating components, AOX and NDs, to the consumption of excessive reducing equivalents and avoidance of ROS and RNS production for maintaining mitochondrial homeostasis is effective in recovering from post-anoxic stress. However, the activation of these components would be futile in O_2_ consumption and energetically burdensome under hypoxic condition. As some non-phosphorylating components are strongly co-expressed, precise coordination between the expressions and activities of these mitochondrial components can provide flexible ATP production and maintain cellular homeostasis in wetland species under more severe hypoxic and anoxic stresses.

The two strategies, LOES and LOQS, in wetland species reflect the different responses to O_2_ deficiency and ATP production at subsequent post-hypoxic and anoxic stresses in their habitats. The difference in strategies could be due to the difference in the requirement of the reserved carbohydrates during the stress condition. Both LOES and LOQS in a single species are also reported. In wetland species with LOES, the developed aerenchyma and high O_2_ supply system by the pressurized gas flow are effective in maintaining high O_2_ availability to the roots, but not all plants have these functions. Further research on the interaction among hormones, O_2_ availability, and primary metabolites is needed to understand their optimal balance among growth, escape, and quiescence to facilitate survival in their habitats. At low O_2_ conditions, the efficient respiratory O_2_ consumption in the roots of wetland species is carried out in soils with NH_4_^+^ as the sole N source because these species can utilize NH_4_^+^ without ARI. Some wetland species in which nitrification occurs due to their high O_2_ supply system can also efficiently use the soil NO_3_^−^. The differences in the preference for N sources among wetland species could also be ascribed to the differences in their anaerobic ATP production systems. The species with high ability of NO_3_^−^ utilization can use the NO_2_^−^-driven ATP production system, and the species specialized for NH_4_^+^ utilization can use the fermentation system. The different N utilization strategies for ATP production may be functionally linked to hypoxia tolerance in wetland species. Thus, further exploration of the ecophysiological mechanisms of aerobic and anaerobic respiratory responses to the N sources in roots of wild wetland species is needed to completely understand the anaerobic stress before global climate change makes the stress more severe.
